# Effective short-term forecasts of Saudi stock price trends using technical indicators and large-scale multivariate time series

**DOI:** 10.7717/peerj-cs.1205

**Published:** 2023-01-06

**Authors:** Ahmad O. Aseeri

**Affiliations:** Department of Computer Science, College of Computer Engineering and Sciences, Prince Sattam Bin Abdulaziz University, Al-Kharj, Saudi Arabia

**Keywords:** Multivariate time series, Time series forecast, Gated recurrent units, Stock market forecast, Multi step forecast

## Abstract

Forecasting the stock market trend and movement is a challenging task due to multiple factors, including the stock’s natural volatility and nonlinearity. It concerns discovering the market’s hidden patterns with respect to time to enable proactive decision-making and better futuristic insights. Recurrent neural network-based methods have been a prime candidate for solving complex and nonlinear sequences, including the task of modeling multivariate time series forecasts. Due to the lack of comprehensive and reference work in short-term forecasts for the Saudi stock price and trends, this article introduces a comprehensive and accurate forecasting methodology tailored to the Saudi stock market. Two steps were configured to render effective short-term forecasts. First, a custom-built feature engineering streamline was constructed to preprocess the raw stock data and enable financial-related technical indicators, followed by a stride-based sliding window to produce multivariate time series data ready for the modeling phase. Second, a well-architected Gated Recurrent Unit (GRU) model was constructed and carefully calibrated to yield accurate multi-step forecasts, which was trained using the recently published historical multivariate time-series data from the primary Saudi stock market index (TASI index), in addition to being benchmarked against a suitable baseline model, namely Vector Autoregression Moving-Average with Exogenous Regressors (VARMAX). The output predictions from the proposed GRU model and the VARMAX model were evaluated using a set of regression-based metrics to assess and interpret the model precision. The empirical results demonstrate that the proposed methodology yields outstanding short-term forecasts of the Saudi stock price trends price compared to existing efforts related to this work.

## Introduction

The stock market is considered one of the most highly complex financial systems as it holds diversified and intricate (macro and micro) factors accountable for the rapid fluctuation of stock prices. The stock price forecast is the task of predicting future prices and movements through analyzing and discovering the hidden patterns of discrete-time historical data using statistical and machine learning methods. Accurate stock price prediction is one of the most challenging tasks in predictive modeling due to the high sensitivity and natural nonlinearity associated with the stock data, making it crucial to be thoroughly addressed through well-processed time-series data to yield informative and constructive forecasts.

Predicting future stock prices and movement has been widely researched in the literature. Some of these works, including Mehmood, Mehmood & Mujtaba (2012), Jarrett (2008), Gilmore & McManus (2003), Fama (1995), Shen & Shen (2016), Al-Shiab (2006), Junior, Salomon & de Oliveira Pamplona (2014), Wadi et al. (2018), Jarrett & Kyper (2011), Banerjee (2014), Almasarweh & Alwadi (2018), Ariyo, Adewumi & Ayo (2014), Mekayel Anik, Shamsul Arefin & Ali Akber Dewan (2020), Siew & Nordin (2012), Cakra & Trisedya (2015), Gharehchopogh, Bonab & Khaze (2013), have applied classical linear methods such as linear regression (Seber & Lee, 2012), random walk (Lawler & Limic, 2010), and Autoregressive Integrated Moving Average (ARIMA) (Zhang, 2003). With the recent improvement of Artificial Intelligence and increased computational capabilities, many others, including Pang et al. (2020), Bing, Hao & Zhang (2012), Yetis, Kaplan & Jamshidi (2014), Mizuno et al. (1998), Chen, Zhou & Dai (2015), Pawar, Jalem & Tiwari (2019), Selvin et al. (2017), Althelaya, El-Alfy & Mohammed (2018), Selvamuthu, Kumar & Mishra (2019), have employed cutting-edge machine learning-based methods such as artificial neural networks (ANN) (McCulloch & Pitts, 1943), Long short-term memory (LSTM) (Hochreiter & Schmidhuber, 1997), and convolutional neural network (CNN) (LeCun & Bengio, 1998). Most of these works study stock forecasting capabilities using univariate time series data by employing the close price as the target feature. Recent advances in time series-based data collection have enabled the vast availability of high-dimensional time series data, facilitating the use of multivariate time series in forecast modeling to render better and possibly more accurate forecasting results. Accordingly, this work considers multivariate-based time series data using historical stock market data featuring various attributes and related characteristics.

An accurate and reliable method for Saudi stock price forecasts which employs large-scale historical data has not been introduced in the literature yet, despite a few efforts reported by Jarrah & Salim (2019), Alamro, McCarren & Al-Rasheed (2019), Alotaibi et al. (2018) and Alsubaie, El Hindi & Alsalman (2019), which involved (a) operating on a minimal subset of the Saudi stock market data, (b) lacking appropriate representation, or (c) reporting results using incorrect metrics. First, the forecasting method presented by Jarrah & Salim (2019) is restricted to predicting the next day’s closing price using a small subset of the Saudi stock historical data from 2011 to 2016. Second, the results reported by Alotaibi et al. (2018) are inaccurate as it uses incorrect evaluation metric (percentage accuracy is not the correct metric for regression problems). Furthermore, the work by Alsubaie, El Hindi & Alsalman (2019) tries to solve a different problem than forecasting, where the authors proposed a cost-sensitive naive Bayes-based classification model to improve the investment return applied to a subset of Saudi stock market data from 2015 to 2018. Finally, Alamro, McCarren & Al-Rasheed (2019) presented a customized methodology that engages sentiment analysis of financial news with recurrent neural network-based modeling to predict the Saudi stock market index using a multivariate time series analysis. Besides reporting forecasts for the next day’s closing price only, we found this study unrepresentative and opaque in the following areas: (1) there is not enough information on how the input stock data was initially collected and used for the forecasting task, and (2) it lacks a clear explanation of how time series stock data was preprocessed and how the forecasting results are related to a company or sector.

The main research question to be investigated by this article is whether the recurrent neural networks (RNN) yield robust and reliable performance in short-term closing price forecasts of the Saudi stock market. Since the related published articles on this topic are either technically restricted or have been incorrectly reported, this article presents a practical and comprehensive methodology for multi-step forecasts of the stock’s closing price, rather than a one-step (*i.e*., next day) forecast which may not be insightful for long-term strategies and planning. The proposed methodology in this work applies a cutting-edge RNN-based learning method, namely Gated Recurrent Units (GRUs), to construct a well-architected forecasting model using the most recent published multivariate time series data of the primary Saudi stock market index, which was made publicly attainable from the Saudi Stock Exchange (Tadawul) (TASI, 2020). The GRU model was trained to produce short-term forecasts for the next five business days and benchmarked against Vector Autoregression Moving-Average with Exogenous Regressors (VARMAX) model, which was also customized to yield short-term forecasts. Furthermore, custom-tailored feature engineering pipeline is made to preprocess the input raw stock data and enable inducing financial-related attributes, followed by a stride-based sliding window to yield trainable short-term multivariate-based time series data ready for the modeling phase. The empirical results interpreted and evaluated by a set of regression-related metrics demonstrate outstanding results with the lowest error rates compared to existing works near this topic, manifesting the proposed methodology’s capability to produce accurate short-term closing price forecasts per company in the Saudi stock market. Simply put, the contribution of this work can be summarized in three folds:

1. Introducing an AI-powered comprehensive forecasting methodology to enable short-term closing price forecasts of the Saudi stock market index assessed using a set of regression-based metrics to interpret the models’ prediction confidence concerning the stock market trends application.
2. We propose a carefully-engineered Gated Recurrent Unit (GRU) model constructed and carefully calibrated to enable precise short-term forecasts.
3. We apply large-scale multivariate time series data of a recently published Saudi stock market index data by TASI (2020), where some stocks have been recorded since 2000, to improve the reliability of the trained model in producing short-term forecasts.

This work attempts to present the best possible AI-assisted decision-making Saudi stock market’s closing price forecasting method, potentially for a deployable solution in real-world settings. The rest of this article is organized as follows: Section 2 presents a literature review of the related works in the stock market predictions. Section 3 presents brief preliminaries about the essential elements used in this article. Section 4 provides a description of the datasets employed during the experiment, followed by demonstrating the proposed preprocessing and modeling method. The article’s experiment is presented in Section 5 followed by a thorough discussion of the results in Section 6. The article is finally concluded in Section 7.

## Literature review

Studies of forecasting the stock market movement and prices have been rapidly growing in recent years, mainly due to the advancement in machine learning-based methods. Chen, Leung & Daouk (2003) attempted to model and predict the direction of the Taiwan Stock Exchange Index by employing the probabilistic neural network (PNN) method with investment strategies. The performance of the proposed modeling design was compared with that of a parametric forecasting approach, namely the generalized methods of the GMM-Kalman filter and random walk forecasting models. The empirical results showed that PNN demonstrates a more substantial predictive capability than the GMM-Kalman filter and the random walk prediction models. Guresen, Kayakutlu & Daim (2011) used a customized artificial neural network (ANN) based model to forecast the stock market index applied to the NASDAQ index forecasts. While the results are not effective enough, they successfully outperformed the existing methods at that time. Another similar research was conducted by Kara, Boyacioglu & Baykan (2011) which attempted to develop two efficient models in predicting the direction of movement in the Istanbul Stock Exchange (ISE) Index using the support vector machine and artificial neural networks, indicating that the average performance of the ANN-based model (75.74%) was significantly better than that of the SVM-based model (71.52%). Li et al. (2011) studied the effect of combining information hidden from the market news with the stock price applied to the Hong Kong stock market use case. The authors utilize the multi-kernel learning (MKL) technique that integrates news articles and short-time history prices, followed by a modeling step using the support vector machine (SVM). The results have shown that the multi-kernel-based model could better use information in news articles and history prices than the model merely combining features of news articles and prices. Li, Dagli & Enke (2007) proposed a reinforcement-oriented forecasting scheme for forecasting short-term stock price movements. The scheme applies actor-only and actor-critic reinforcement learning, respectively, and is eventually compared with a supervised-only model and classical random walk benchmark. The actor-critic-based system’s performance was better than other alternatives, while the actor-only system also showed efficacy.

Sun et al. (2019) introduced a data-driven approach that combines classical financial pricing models with the artificial neural networks, named ARMA-GARCH-NN, that incorporate unique designs of feature selection, cross-validation, and ensemble learning to enable capturing intra-day patterns for stock market shock forecast. The authors investigate the value of historical information in capturing unexpected market movements, defined as the innovation obtained from ARMA-GARCH models. The empirical studies applied to high-frequency data of the U.S. stock market show that (i) ARMA-GARCH-based market shocks at the intra-day level are predictable, and (ii) the predicted market shocks can serve as new trading signals to benefit the decision-making process for financial investments. Moreover, Cao, Li & Li (2019) investigated the impact of noise inherent in the non-linear and non-stationary stock market data by introducing two-hybrid forecasting models that combine the empirical mode decomposition (EMD) and its advanced version CEEMDAN with the long short-term memory (LSTM) to predict the financial time series. The idea is to decompose the original time series into several sub-series under different frequencies using the EMD and CEEMDAN methods, and the sub-series are respectively predicted as the input data of the LSTM model. Finally, all the predictions are re-constructed to get the final result. The proposed method is tested using four major global stock indices. The results are compared with a single LSTM, support vector machine (SVM), multi-layer perceptron (MLP), and other hybrid models, demonstrating a better performance in one-step-ahead forecasting of financial time series. Chen, Lin & Wang (2019) studied the problem where traditional statistical methods cannot extract relevant features for mining the financial time series data. Thus, the authors proposed a dual-phase trend prediction model (TPM) based on dual features and a dual attention mechanism to predict the direction and duration of stock price changes. The performance of the proposed method outperforms the state-of-art methods, including SVR, LSTM, CNN, LSTM-CNN, and TPM-NC, under various training and testing parameters with different stock index data sets. Finally, Javed Awan et al. (2021) moves toward the financial big data problem where modeling is challenging. The authors implemented several machine learning models using the Spark MLlib library in PySpark to predict market trends using the stocks of several top companies containing 10-year historical data to predict stock price movements. Experimental results indicated that linear regression, random forest, and generalized linear regression deliver 80–98% accuracy.

## Preliminaries

Before describing the proposed methodology, we need to establish an understanding of the essential elements involved in this experiment, including the definition of multivariate time series and their special properties with forecasting strategies, problem formulation, and the RNN-based modeling component.

### Time series forecasting

Time series is a collection of sequential vector-valued variables of some phenomenon indexed over time. Time series forecasting is the task of predicting future values of given time-series observations by analyzing historical time series data to check for patterns of time decomposition, such as trends, seasonal patterns, cyclic patterns, and regularity. Univariate time series implies that forecasting is based on a single variable of time series observations without considering the effect of the other variables, making them desirable for computational efficiency especially when data availability is already scarce. On the other hand, multivariate time series have more than one time-dependent variable where each variable depends not only on its past values but also tends to have some interdependency with other variables. Although the multivariate time series setting is known for difficulty in extracting beneficial information for the prediction process (Chayama & Hirata, 2016), informative forecasts are often assumed to be conveyed by multivariate time series since advanced statistical methods can leverage the dependencies between variables to improve the forecasting accuracy (Yin & Shang, 2016; Zhang et al., 2017; Lang et al., 2019).

### Problem definition

Multivariate time series forecasting has numerous valuable applications, such as predicting electricity consumption, solar power production, and stock trend movements. The potential interdependency among variables in the multivariate-based time series can lead to better and more coherent learning results, especially when variables are highly correlated. This research seeks to employ the multivariate-based time series analysis to enable better information extraction about the data and their underlying dynamics to improve the short-term prediction accuracy.

#### Problem formulation

Given a set of observed time series $X = \{ {x_1},{x_2}, \cdots ,{x_n}\}$, where ${x_t} \in {{\mathbb R}^d}$ is a real vector of dimension $d$ representing an observed time serie at time $t$, the goal of multivariate time series forecasting is to forecast the future time, ${x_{t + h}}$, where $h$ is the desirable fixed-value horizon ahead with respect to different tasks.

The horizon length $h$ is specified based on multiple parameters, including the data and environment settings. For instance, the choice of the horizon $h$ for stock market data can be anywhere from minutes to days, as this range makes sense for the stock market domain.

### VARMA with exogenous regressors

The Vector Autoregressive Moving-Average (VARMA) model extends the ARMA/ARIMA model to work with time series with multiple response variables (vector time series). It is one of several statistical analyses frequently used for multivariate-based time series studies. The model includes the dynamic relationship between the multiple response variables as well as the relationship between the dependent and independent variables. It is a combination of Vector Auto-Regressive (VAR) and Vector Moving-Average (VMA) models that supports the multivariate time series modeling by considering both the lag order (p) and order of moving average (q) in the model. The VARMA model can operate as a VAR model by just setting the q parameter to 0, and it also can operate like a VMA model by just setting the p parameter to 0. Moreover, the Vector Autoregression Moving-Average with Exogenous Regressors (VARMAX) is an extension of the VARMA model that includes modeling with exogenous variables (also called covariates). In practice, the target series is referred to as an endogenous sequence to contrast from exogenous sequences that are included directly in the model at each time step but not modeled in the same way as the target endogenous sequence. Like VARMA, VARMAX can also operate as VARX and VMAX to model the subsumed models with exogenous variables.

Formally, the endogenous vector ${{\bf y}_t}$ is said to follow a vector autoregressive moving average with exogenous variables $VARMAX(p,q,s)$ if it satisfies an equation of the form (Östermark, 1994; Kadiyala & Kumar, 2014; SAS, 2016):

(1) $${{\bf y}_t} = \sum\limits_{i = 1}^p {{\Phi _i}} \; {{\bf y}_{t - 1}} + \sum\limits_{i = 0}^s {\Theta _i^ * } \; {{\bf x}_{t - 1}} + {{\bf \epsilon }_t} - \sum\limits_{i = 1}^q {{\Theta _i}} \; {\varepsilon _{t - 1}}$$

where the output variables of interest, ${{\bf y}_t} = ({y_{1t}}, \cdots ,{y_{kt}})$ referred to response or endogenous variables, can be influenced by other input variables, ${{\bf x}_t} = ({x_{1t}}, \cdots ,{x_{mt}})$ referred to as regressor or exogenous variables. The unobserved noise variables, ${\varepsilon _t} = ({\varepsilon _{1t}}, \cdots ,{\varepsilon _{kt}})$, are a vector white noise process. Therefore, the model $VARMAX(p,q,s)$ can be written as:

(2) $$\Phi (B){{\bf y}_t} = {\Theta ^ * }(B){{\bf x}_t} + \Theta (B){\varepsilon _t}$$

where,

$\eqalign{& \Phi (B)\,\,\, = {I_k} - {\Phi _1}B - \cdots - {\Phi _p}{B^p} \cr & {\Theta ^ * }(B)\,\,\, = \Theta _0^ * + \Theta _1^ * B + \cdots + \Theta _s^ * {B^s} \cr & \Theta (B)\,\,\, = {I_k} - {\Theta _1}B - \cdots - {\Theta _q}{B^q}}$

are matrix polynomials in *B* in the backshift operator, such that ${B^i}{{\bf y}_t} = {{\bf y}_{t - 1}}$, the ${\Phi _i}$ and ${\Theta _i}$ are $k \times k$ matrices, and $\Theta _i^ *$ are $k \times m$ matrices. For the sake of achieving the complete definition, the following assumptions are made:

-
$E({\varepsilon _t}) = 0$, $E({\varepsilon _t}\varepsilon _t^\prime ) = \sum$ which is positive-definite, and $E({\varepsilon _{\bf t}}\varepsilon _{\bf s}^\prime ) = 0$ for $t \ne s$.
- For stationarity VARMAX, the roots of $|\Phi (z)| = 0$ and $|\Theta (z)| = 0$ are outside the unit circle.
- The exogenous independent variables ${{\bf x}_t}$ are not correlated with residuals ${\varepsilon _t}$, $E({{\bf x}_t}\varepsilon _t^\prime ) = 0$.
- The exogenous variables can be stochastic or non-stochastic.

### Gated recurrent units

We first present a brief exposition of recurrent neural networks (RNNs). RNN, first conceived by Elman (1990), is an extension of the conventional feedforward neural network used for sequence modeling problems. RNNs can be seen as a universal approximator for dynamical systems of variable-length input sequences (Schäfer & Zimmermann, 2006). RNNs can let information persist within the network over time, making them suitable for sequence learning modeling. Formally, given a sequence ${\bf X} = ({x_1},{x_2},...,{x_t})$, a default RNN updates a recurrent hidden state ${{\bf h}_t}$ at time $t$ as (Olah, 2015):

(3) $${{\bf h}_t} = f({{\bf h}_{t - 1}},{x_t})$$

where $f$ is commonly chosen as a nonlinear activation function, such as sigmoid or tanh, with an affine transformation of both ${h_{t - 1}}$ and ${x_t}$. Thus, the update of the recurrent hidden state can be rewritten as:

(4) $${{\bf h}_t} = \Phi ({W_{{x_t}}} + \Theta {{\bf h}_{t - 1}} + b)$$

where ${x_t}$ is $m$-dimensional input vector at time $t$, ${{\bf h}_t}$ is $n$-dimensional hidden state, $\Phi$ is a nonlinear activation function, *W* is weight matrix of size $n \times m$, $\Theta$ is recurrent weight matrix of size $n \times n$, and $b$ is a bias of size $n \times 1$.

The default RNN is deficient in capturing long-term dependencies because the gradients tend to either vanish or explode with long sequences (Bengio, Simard & Frasconi, 1994). Recall a well-known phenomenon in canonical recurrent neural networks (RNNs) called long-term dependencies (Bengio, Simard & Frasconi, 1994), in which a system’s desired output at time *T* depends on inputs presented at times $t < T$. In order words, as the sequence length increases during training, the gradients tend to vanish, leading to the problem of vanishing gradients. Vanishing gradients prevent the network from learning the underlying dynamics and hidden patterns extracted from the long-term dependencies, which is beneficial in producing accurate future forecasts. Vanishing gradients also arise when the network dynamics have attractors necessary to store bits of information over the long term (Chandar et al., 2019). Successful architectures, such as LSTM and GRU, alleviate this issue by introducing memory in the form of information skipped forward across transition operators, with gates bounded by activation functions (sigmoid, tanh) to determine which information to skip, releasing the hidden states (*i.e*., the memory) from their output states to reduce the vanishing problem.

**Figure 1:**
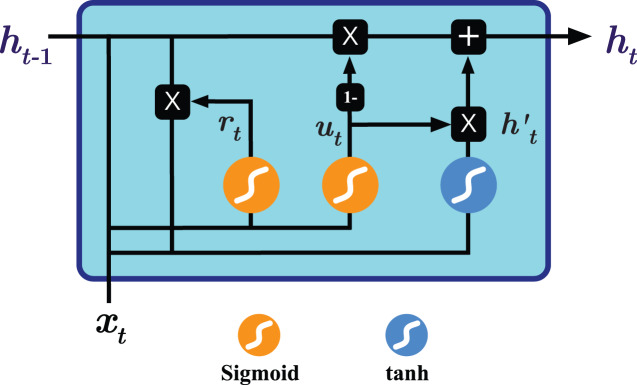
Illustration of the Gated Recurrent Unit (GRU) architecture.

To overcome the shortcoming mentioned above, two RNN-based variants have been introduced, namely Long Short Term Memory (LSTM) (Hochreiter & Schmidhuber, 1997) and Gated Recurrent Units (GRU) (Chung et al., 2014). Both variants seek to keep long-term dependencies effective while alleviating the vanishing and exploding gradient problems (Chandar et al., 2019; Aseeri, 2021). As seen in Fig. 1, GRU operates on two gates: reset and update gates. The architecture essentially combines the forget and input gates used by Long term short memory (LSTM) into the *update gate* and merges the cell state and hidden state into the *reset gate*, resulting in a simpler design and consequently lower number of trainable parameters needed compared to the LSTM variant, improving the training time and performance (Chung et al., 2014). The operations performed by the GRU model are as follows (Chung et al., 2014; Dey & Salem, 2017):

(5) $$\eqalign{& {r_t}\,\,\, = \sigma ({W_r} \cdot [{{\bf h}_{t - 1}},{x_t}]) \cr & {\theta _t}\,\, = \sigma ({W_u} \cdot [{{\bf h}_{t - 1}},{x_t}]) \cr & {{{\bf {h}^{\prime}}}_t}\,\, = tanh(W \cdot [{r_t} * {{\bf h}_{t - 1}},{x_t}]) \cr & {{\bf h}_t}\,\, = (1 - {\theta _t}) * {{\bf h}_{t - 1}} + {\theta _t} * {{{\bf {h}^{\prime}}}_t}}$$

where ${r_t}$ and ${\theta _t}$ are respectively the reset and update gates at time $t$, ${W_ * }$ are weight matrices, and $\cdot$ denotes component-wise multiplication.

## Methodology

### Data description and exploration

**Figure 2:**
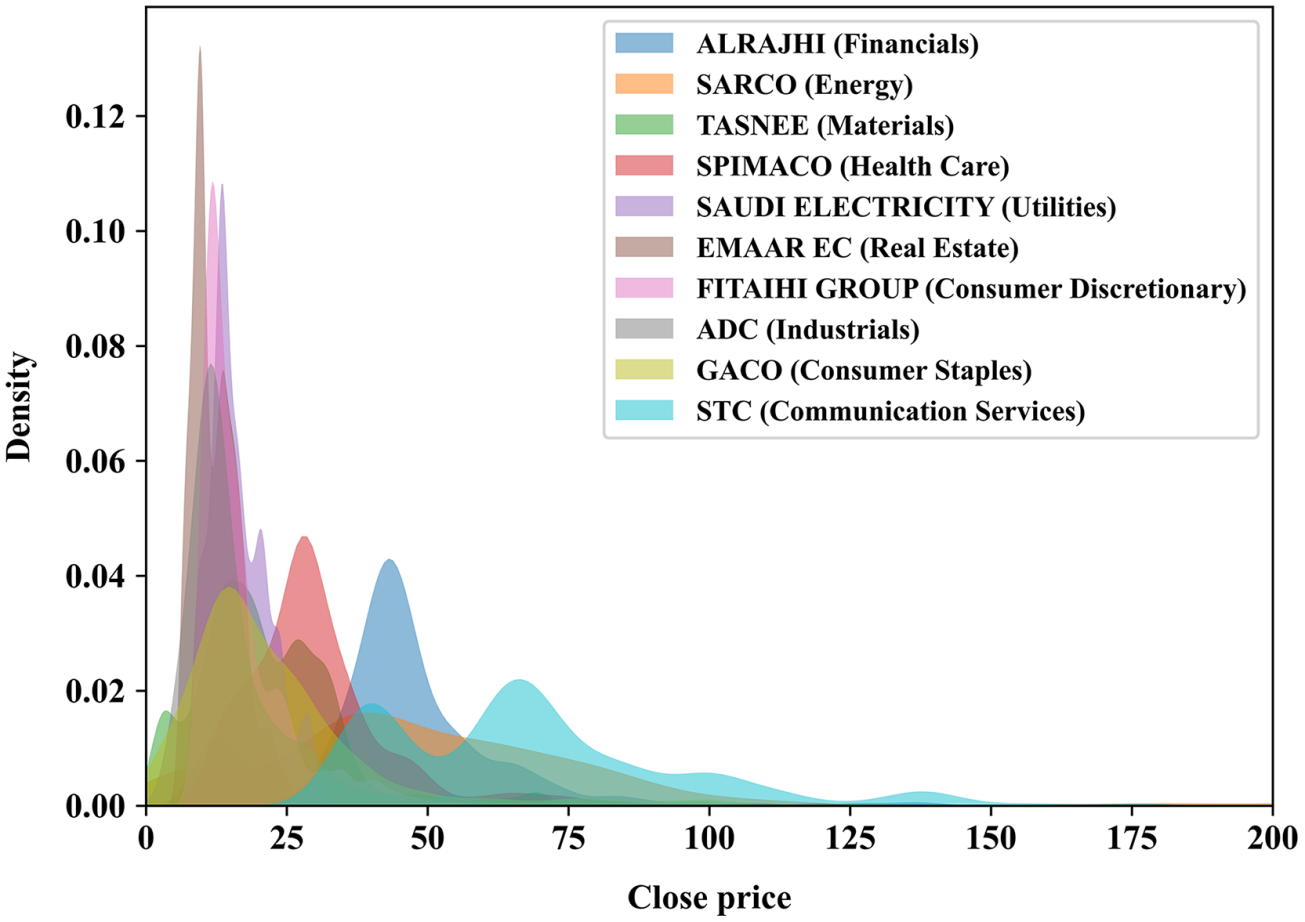
KDE plot illustrating the analytical distribution of the closing price of each of the 10 companies chosen randomly in this experiment. A company’s sector is stated within the parentheses.

To achieve the best possible forecasting results, especially in the time-series domain, it is important to employ long historical and well-preprocessed time series data to help capturing the hidden patterns for the domain of interest, such as stocks’ trends and movement. In this experiment, we use a recently published multivariate time series of the primary Saudi stock market index, particularly the Tadawul All Share Index (TASI), made freely accessible by TASI (2020). The TASI dataset represents daily closing prices of TASI index companies, which are distributed into ten sectors featuring 14 attributes as shown in Table 1 where eight of which are numerical features: open, high, low, change, percentage change, volume traded, value traded, and number of trades. Since the Saudi stock market index is as old as 2001, some companies in the dataset have been recorded since 2001, while others have recently joined the market, meaning that the recording data of a new company begins immediately starting from the joining date. We randomly selected ten companies, one from each sector, to be modeled by our proposed method and listed in Table 2. In addition, Fig. 2 illustrates the distribution of the closing price of each of the ten chosen companies.

**Table 1:** Feature descriptions of the dataset used in this experiment.

Feature	Type	Description
symbol	Integer	The symbol or the reference number of the company
name	String	Name of the company
trading_name	String	The trading name of the company
sectoer	String	The sector in which the company operates
date	Date	The date of the stock price
open	Numeric	The opening price
high	Numeric	The highest price of the stock at that day
low	Numeric	The lowest price of the stock at that day
close	Numeric	The closing price
change	Numeric	The change in price from the last day
perc_change	Numeric	The percentage of the change
volume_traded	Numeric	The volume of the trades for the day
value_traded	Numeric	The value of the trades for the day
no_trades	Numeric	The number of trades for the day

**Table 2:** A list of randomly selected companies involved in this study, each representing a sector.

Sector	Company name	Trading name
Financials	Al Rajhi Bank	ALRAJHI
Energy	Saudi Arabia Refineries Co.	SARCO
Materials	National Industrialization Co.	TASNEE
Health care	Saudi Pharmaceutical Industries and Medical Appliances Corp.	SPIMACO
Utilities	Saudi Electricity Co.	SAUDI ELECTRICITY
Real estate	Emaar The Economic City	EMAAR EC
Consumer discretionary	Fitaihi Holding Group	FITAIHI GROUP
Industrials	Al-Ahsa Development Co.	ADC
Consumer staples	Al Gassim Investment Holding Co.	GACO
Communication services	Saudi Telecom Co.	STC

### Temporal multivariate processing

**Figure 3:**
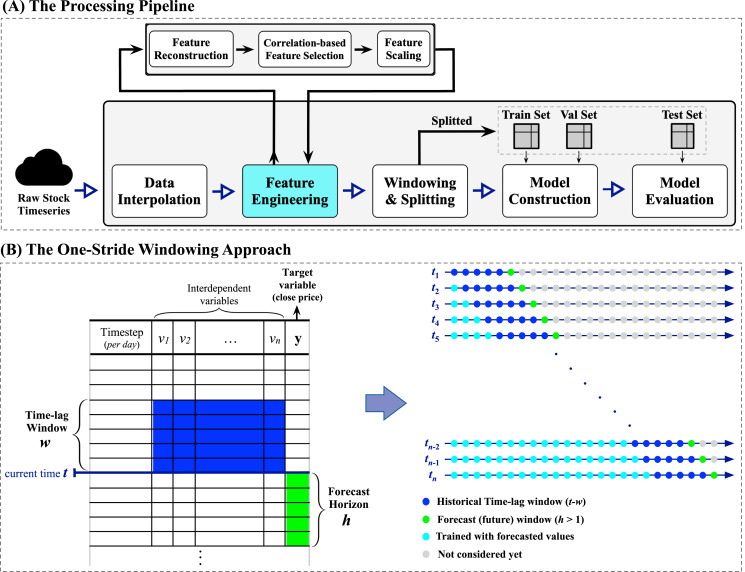
A visual representation of the proposed forecasting system. The top subfigure (A) represents the processing pipeline, while the bottom subfigure (B) illustrates the one-stride windowing approach used with the input datasets.

In this study, the process of Saudi stock trend prediction is considered a regression problem where the goal is to produce future horizon closing price forecasts of size $h$, where $h$ is set to five representing the five trading days, given a past window of multivariate time-lagged historical time series^1^ 1The selection of *h* = 5 is because the stock trading in Saudi Arabia is five business days per week.. Moreover, this work explores the performance of the proposed prediction system when employing a number of financial-related technical indicators to improve forecasts. To the best of our knowledge, this is the only work reporting multi-step closing price forecasts of the Saudi stock TASI index. The proposed methodology is visualized in Fig. 3, which depicts the processing pipeline (top subfigure) and the windowing approach applied to the raw time series data (bottom subfigure).

The forecasting pipeline shown in the top subfigure of Fig. 3 is tailored to ensure systematic and sound preprocessing and modeling. The pipeline involves five essential modules, each performing a specific task. The process begins with initiating a streaming data reader from the raw stock data to enable retrieving multivariate time series data. When a specific company’s multivariate time series is fully retrieved, the data interpolation module begins cleaning the retrieved multivariate time series by (1) removing duplicate timesteps when detected and (2) performing linear interpolation using the forward technique by replacing missing values with the last valid observation propagated to fill the consecutive gaps (the missing values are the weekend days as the market is closed). Next, the feature engineering module is responsible for conducting three major processes: (i) feature reconstruction, (ii) correlation-based feature selection, which will be elaborated in detail in the next subsection “Feature Engineering: Technical Indicators and Feature Selection”, and (iii) the min-max normalization. After that, the windowing and splitting module performs a customized one-stride sliding window procedure to form adequate sequences for the modeling phase, followed by splitting the overall sequences into three sets, namely training, validation, and testing sets. Here, the word *stride* implies the amount of movement/shift to the right direction in a time, so one-stride means one movement to the right at a time. Looking closely at the left side of the windowing approach subfigure in Fig. 3, an iterative top-down procedure applied to the multivariate time series data generates multivariate sequences with a vector of future horizons, thus constructing supervised-like multistep sequences ready for training and validating the forecasting model. For instance, at a timestep $t$, a one-stride sliding window is rolled to specify a batch that involves the historical time-lag window of size $w$ (in blue) and the forecast window of size $h$ (in green). Consequently, his process can be formally expressed as:

${y_{t + h}} = {f_h}\{ {y_t}, \ldots ,{y_{t - w}}\}$

where $h$ is the number of steps to forecast into the future (*i.e*., the desirable future horizons), $w$ is the size of the time-lag window, $t$ is the timestep (*i.e*., the day), and ${f_h}$ is any arbitrary learner. Furthermore, it is well-recognized that the most critical parameter in multivariate time series forecasting is the proper determination of the number of past observations, *i.e*., the time-lag window. Thus, we performed a trial and error method with different sizes of multivariate time-lag windows, including 1, 5, 10, and 15 days, to choose the best possible window size that yields better performance. We concluded that the time-lag window of size 5 days (*i.e*., $w = 5$) works well in providing satisfactory forecasting results while maintaining an efficient processing time. Finally, both model construction and model evaluation modules will be elaborated on in the upcoming subsection “Modeling Design”.

### Feature engineering: technical indicators and feature selection

Technical indicators (TIs) are heuristic pattern-based indicators produced by a feature of interest, such as the price, volume, *etc*., which are usually utilized as exogenous features to aid in identifying possible hidden patterns, trends, and movements within the historical time series. In practice, the success of employing the technical indicators relies heavily on the availability of sufficient historical data, making them conceivably applicable in this experiment. In this study, we plan to explore the effectiveness of employing technical indicators as additional exogenous features to the input multivariate TASI index data. We hypothesized that we would get enhanced forecasting results when exploiting technical indicators as they can improve the interdependency between the exogenous features with respect to the endogenous target variable, hence better capturing hidden patterns and trends within the historical time series.

Accordingly, we perform *feature reconstruction* within the feature engineering module where the original features, listed in Table 1, are extended using an arbitrary set of financially-related technical indicators, computed with respect to the target feature of interest in this study, *i.e*., the close price. We choose five essential and widely adopted technical indicators in finance, namely Weighted Moving Average (WMA), Double Exponential Moving Average (DEMA), Triple Exponential Moving Average (TEMA), Hull Moving Average (HMA), and Zero-Lag Exponential Moving Average (ZLEMA), thus resulting fourteen features in total (nine original numerical features, five technical indicators features). The calculation is carried out in two steps: (1) calculating a new vector of attributes on each trading day, representing the values of the selected technical indicators where the input parameter lag in each indicator is set to 5, and (2) extending the original exogenous features with the new computed vector. For the sake of completeness, we show the formulations of the indicators mentioned above as follows: if $n$ is the number of periods, $w$ is the weighting factor, $\alpha$ is a smoothing factor, and ${x_t}$ is the data observation at the period $t$, the well-known Weighted Moving Average (WMA) is defined as (Perry, 2010):

(6) $$WMA = \displaystyle{{\sum\nolimits_{t = 1}^n {{w_t}} {x_t}} \over {\sum\nolimits_{t = 1}^n {{w_t}} }}$$

also, the Double Exponential Moving Average (DEMA) and Triple Exponential Moving Average (TEMA) are defined as (Rozario et al., 2020):

(7) $$\matrix{ {DEMA} \hfill & { = 2 \times EMA - EMA(EMA)} \hfill \cr }$$

(8) $$\matrix{ {TEMA} \hfill & { = (3 \times EMA - 3 \times EMA(EMA)) + EMA(EMA(EMA))} \hfill \cr }$$

where,

$\matrix{ {EM{A_t}} & { = \left\{ {\matrix{ {{x_0} }\hfill  & {t = 0} \cr {\alpha {x_t} + (1 - \alpha )EM{A_{t - 1}}} & {t > 0} \cr } } \right.} \cr }$

the Hull Moving Average (HMA) makes a moving average more responsive while maintaining a curve smoothness. It uses a combination of three WMAs with different sizes of period, and it is computed as Hull (2005):

(9) $$HMA = WM{A_{\sqrt n }}(2 \times WM{A_{\displaystyle{n \over 2}}}(X) - WM{A_n}(X))$$

finally, the Zero-Lag Exponential Moving Average (ZLEMA) modifies the Exponential Moving Average (EMA) to greatly reduce lag. It is computed as (Ehlers & Way, 2010):

(10) $$ZLEMA = K(2 \times {x_0} - {x_{ - lag}}) + (1 - K)ZLEM{A_{ - 1}}$$

where,

$\matrix{ K \hfill & { = \displaystyle{2 \over {(n + 1)}}} \hfill \cr {lag} \hfill & { = \displaystyle{{(n - 1)} \over 2}} \hfill \cr }$

Next, to ensure that the newly added technical indicators and the original exogenous features have the desired level of relevance and interdependencies, we adopt Pearson’s correlation as a correlation-based feature selection method to return the most highly-correlated features with respect to the target variable. Pearson’s correlation can be utilized to translate the linear relationship’s strength between every two variables. It outputs values in the interval [−1, +1] where +1 implies total positive correlation, −1 total negative correlation, and 0 no correlation. As per the standard literature, for a pair of variables $(X,Y)$, the linear correlation coefficient $r$ is given by:

$r = \displaystyle{{\sum {({X_i} - {{\bar X}_i})} ({Y_i} - {{\bar Y}_i})} \over {\sqrt {\sum {{{({X_i} - {{\bar X}_i})}^2}} } \sqrt {\sum {{{({Y_i} - {{\bar Y}_i})}^2}} } }}$

**Figure 4:**
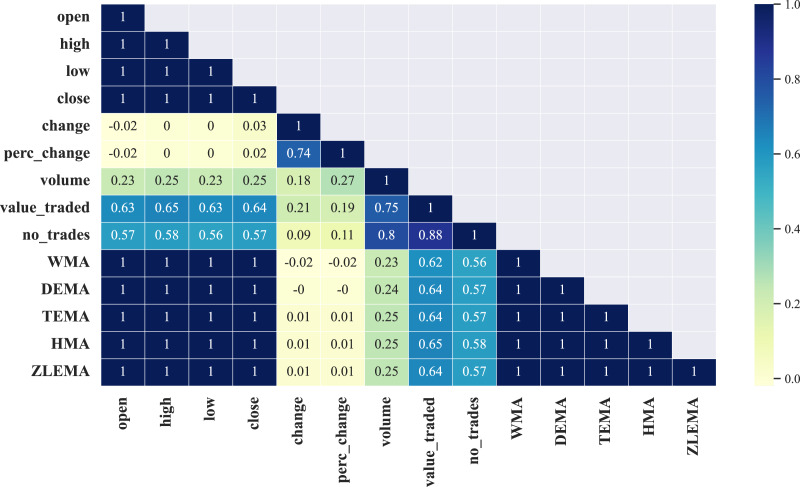
Illustration of the correlation matrix applied on the preprocessed time series of the Saudi Arabia Refineries Co.

This study found that all correlation matrices rendered from the selected ten companies have an identical strong positive correlation between only nine out of the total fourteen features, namely: open, high, low, close, WMA, DEMA, TEMA, HMA, ZLEMA, which can be observed in Fig. 4 where we visualize the correlation matrix (half diagonally since it is symmetric) from a selected company, *i.e*., the Saudi Arabia Refineries. The rest of the correlation matrices of the remaining eight companies involved in this work are shown in Fig. A1 in the Appendix.

### Modeling design

**Figure 5:**
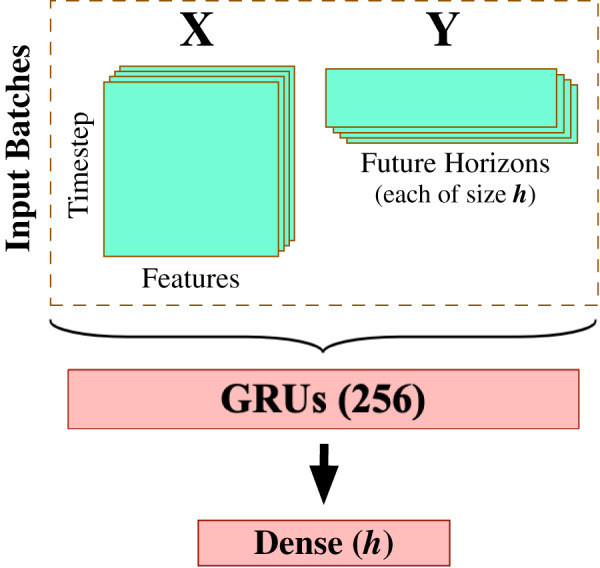
GRU-based model architecture.

After completing the data preprocessing phase, we begin modeling the processed multivariate time series data using a carefully-engineered Gated Recurrent Units (GRU) model. This work deliberately applies the gated recurrent units method for multivariate time series forecasting as one of the most effective RNN-based methods that provide accurate results with efficient computation. We spent quite some time experimenting with varying design architectures and hyperparameter tuning options to yield the best possible forecasting results, including Fig. 5 that demonstrates the most suitable model design and configuration given the input preprocessed multivariate time series data. This final architecture involves one GRU layer composed of 256 GRU processing units (see Fig. 1 for depicting the generic GRU processing unit) and followed by a dense layer of *linear* activation units equal to the size of the forecast horizon $h$ (in this work, $h = 5$).

In order to verify the correctness of the GRU forecasting estimates, we need to compare them with a baseline statistical method such as AutoRegressive Integrated Moving Average (ARIMA). Since we operate on the multivariate time series modeling, we employ an ARIMA variant designed for handling exogenous data, called Vector Autoregressive Moving-Average with Exogenous Regressors (VARMAX) (see subsection “VARMA with Exogenous Regressors” for more details about this variant). The comparison results will be illustrated later in the upcoming section “Results and Discussion”.

## Empirical experiment

### Experimental setup

The experimental environment is implemented using the Python programming language (Python 3). The neural network-based modeling framework of choice is TensorFlow v2.4.1. We also implemented the VARMAX modeling with the support of pmdarima API v2.0.0 (Smith, 2017). To ensure sound model training and evaluation, the input time series is splitted into three sets: train set starts from the first-ever trading day until 31-12-2015, the validation set is from 01-01-2016 to 31-12-2016 while the testing set is from 01-01-2017 to 01-04-2020. The experiment was performed on a workstation having Intel Core i7-10700KF (3.8 GHz) CPU, Nvidia Geforce RTX 3060 Ti (8 GB) GPU, and 64 GB of system RAM. For each company involved in this experiment and listed in Table 2, the experiment was run five times, and we reported only the best run.

The neural network-based methods seek to minimize errors between predicted and true values assessed using a suitable cost function by iteratively training and tuning the model’s hyperparameters using a gradient-based descent optimization method. For training the GRU model, we found out that Nesterov-accelerated Adaptive Moment Estimation (NADAM) (Dozat, 2016), with the learning rate of $1 \times {10^{ - 4}}$, works well during the training and the validation of the GRU model design. The mean squared error was appointed as the cost function of choice to estimate the model’s errors, while the batch size fed to the model during the feedforward process was fixed to be 10% of the training data. Furthermore, the activation functions for the GRU gates are left to the conventional default. That is, for both the update and reset gates, the activation is the sigmoid function, while the hyperbolic tangent function (tanh) is used with the recurrent activation gate. For the sake of reproducibility, Table 3 reports the hyperparameters options along with their description of each model used in this experiment.

**Table 3:** Descriptions of the hyperparameters used in this experiment for reproducibility purposes.

Model	Hyperparameter description
**VARMAX**	p, d, q = $(5,0,0)$ iterations = 100 optimizer = Limited-memory BFGS
**GRU**	Optimizer = Nesterov-accelerated Adam (Nadam) learning_rate = $1 \times {10^{ - 4}}$ early_stopping = 20 epochs = 1,000 batch_size = $10\%$ of train data

### Evaluation metrics

The performance of neural network-based methods is generally evaluated in terms of metrics related to the models’ discriminative power. In principle, making a proper and correct selection of evaluation metrics for quantifying prediction performance enable the correct interpretation of the model’s skill and capability in making the forecasts. Therefore, we choose a regression-based metric set that assesses our models’ precision for the multi-step forecasting task. Various evaluation metrics are available for calculating the accurateness of the forecasts, but the selected metrics shown in Table 4 have been the default metrics for regression problems as well as widely adopted for evaluating the performance of time series forecasts. Here, $y$, $x$, and $n$ represent the forecasted value, the actual value, and the number of sample data for the period, respectively.

**Table 4:** Performance evaluation metrics used in this study.

Evaluation metric	Equation
Mean absolute error (MAE)	$\displaystyle{{\sum\nolimits_{i = 1}^n | {y_i} - {x_i}|} \over n}$
Mean squared error (MSE)	$\displaystyle{{\sum\nolimits_{i = 1}^n {{{({y_i} - {x_i})}^2}} } \over n}$
Root mean squared error (RMSE)	$\sqrt {\displaystyle{{\sum\nolimits_{i = 1}^n {{{({y_i} - {x_i})}^2}} } \over n}}$
Coefficient of determination (R^2^)	$1 - \displaystyle{{{\rm sum \;squared \;regression\; (SSR)}} \over {{\rm total \;sum \;of \;squares\; (SST)}}}$

The evaluation metrics shown in Table 4 are commonly used in predictive modeling. That is, mean absolute error (MAE) measures the average error magnitude in a set of predictions using the absolute value. This type of error measurement is proper when measuring prediction errors in the same unit as the original series. The mean squared error (MSE) formula replaces the absolute value with a square, giving large errors a relatively greater influence on MSE than smaller ones. MSE has nice mathematical properties, making it easier to compute the gradients. Similarly, root means squared error (RMSE) measures the average magnitude of the error but with a square root on the average squared differences between prediction and actual observation (MSE and RMSE are monotonically related, *i.e*., through the square root). Finally, the coefficient of determination (R^2^) is the goodness-of-fit measurement of how well the regression line approximates the actual observation at the 0–1 scale (or 0–100%) where a higher value is considered desirable as opposed to the MAE, MSE, and RMSE where lower is deemed desirable. The sum squared regression (SSR) is the sum of the *residuals* squared, and the total sum of squares (SST) is the sum of the distance the data is away from the mean all squared.

## Results and discussion

This section analyzes and demonstrates the performance of our proposed methodology and modeling design discussed in “Methodology”. All results shown in this section are based on a separate test set of preprocessed multivariate-based time series used for neither training nor hyperparameter tuning. The test time series is set from 01-01-2017 to 01-03-2020. To fully evaluate the effectiveness of the resulting forecasting models, we collect the predictions of the five-day horizon period for each out-of-sample observation and evaluate them using the chosen metrics discussed in subsection “Evaluation Metrics”.

Recall that one central theme in this work is to explore the effectiveness of employing technical indicators as additional features incorporated into the multivariate-based TASI dataset to improve the accuracy of the short-term multi-step closing price forecasts (see subsection “Feature Engineering: Technical Indicators and Feature Selection”). We hypothesized that we would get enhanced forecasting results when adding technical indicators to improve the interdependency between the exogenous features, hence better capturing hidden patterns and trends within the historical time series. Afterward, the relevance between the exogenous features is inspected using Pearson’s correlation coefficient as a correlation-based feature selection method to return the most highly-correlated features with respect to the endogenous target variable.

This experiment applies an RNN-based gated recurrent units (GRU) modeling design tailored to obtain the best possible forecasting results using the TASI dataset. We compare the GRU model’s results with the VARMAX model’s results, with the latter being the baseline. To enable the most helpful comparison, we perform two experiments per model: one by operating on the original multivariate time series data, *i.e*., preprocessed version of the original numerical features listed in Table 1, and the other one by the selected feature set resulting from the feature engineering module’s step elaborated previously in subsection “Feature Engineering: Technical Indicators and Feature Selection”, which will be marked with the asterisk (*) symbol in the performance comparison Table 5.

**Table 5:** Performance comparison between the baseline VARMAX model and the proposed GRU model applied to the selected 10 companies listed in Table 2, where the bold cells indicate the better result. The asterisk (*) in front of the model implies that the model was trained on data preprocessed using the proposed feature engineering step discussed in “Feature Engineering: Technical Indicators and Feature Selection”. For all results, the selected sizes of time-lag window and the future horizon were set to and, respectively (i.e., five trading days, see “Temporal Multivariate Processing”).

Company (Sector)	Model	Evaluation metric			
		MAE	MSE	RMSE	R2
AlRAJHI (Financials)	VARMAX	1.0242	2.0669	1.4377	0.9806
	VARMAX*	1.0253	2.07	1.4387	0.9806
	GRU	3.0632	203.4262	13.9463	−0.911
	GRU*	**0.8097**	**1.3925**	**1.1484**	**0.987**
SARCO Co. (Energy)	VARMAX	1.0733	2.6944	1.6415	0.8957
	VARMAX*	1.056	2.6497	1.6278	0.8974
	GRU	1.2986	3.7538	1.9169	0.8547
	GRU*	**0.805**	**1.65**	**1.2403**	**0.9362**
TASNEE Co. (Materials)	VARMAX	0.4295	0.339	0.5822	0.9616
	VARMAX*	0.4285	0.3302	0.5747	0.9626
	GRU	0.4164	0.3201	0.5521	0.9638
	GRU*	**0.3479**	**0.2345**	**0.4719**	**0.973**
SPIMACO Co. (Health Care)	VARMAX	0.5103	0.5645	0.7513	0.9551
	VARMAX*	0.5021	0.5525	0.7433	0.956
	GRU	0.4871	0.4847	0.6822	0.9614
	GRU*	**0.3994**	**0.3678**	**0.5923**	**0.9707**
Saudi Electricity Co. (Utilities)	VARMAX	0.4314	0.3747	0.6121	0.959
	VARMAX*	0.4223	0.3617	0.6014	0.96
	GRU	0.3304	0.2419	0.4815	0.973
	GRU*	**0.3198**	**0.2262**	**0.4636**	**0.975**
EMAAR EC Co. (Real Estate)	VARMAX	0.3035	0.1868	0.4322	0.9793
	VARMAX*	0.2967	0.1851	0.4302	0.9795
	GRU	0.3651	0.2177	0.4535	0.9758
	GRU*	**0.2282**	**0.1187**	**0.3304**	**0.9868**
FITAIHI GROUP (Consumer Discretionar)	VARMAX	0.1852	0.0713	0.267	0.922
	VARMAX*	0.1776	0.0686	0.262	0.925
	GRU	0.1836	0.0615	0.2407	0.9329
	GRU*	**0.1341**	**0.0429**	**0.2016**	**0.953**
ADC Co. (Industrials)	VARMAX	0.2365	0.1295	0.3599	0.94
	VARMAX*	0.2296	0.1231	0.3508	0.943
	GRU	0.2478	0.137	0.3606	0.936
	GRU*	**0.1818**	**0.0832**	**0.2805**	**0.961**
GACO Co. (Consumer Staples)	VARMAX	0.2562	0.1336	0.3655	0.932
	VARMAX*	0.238	0.1232	0.351	0.937
	GRU	0.5177	0.3715	0.5774	0.8095
	GRU*	**0.2488**	**0.1167**	**0.3298**	**0.9401**
STC Co. (Communication Services)	VARMAX	1.7461	6.2713	2.5042	0.969
	VARMAX*	1.7603	6.2301	2.496	0.969
	GRU	6.9844	69.0844	8.2641	0.656
	GRU*	**1.4412**	**4.6063**	**2.0916**	**0.977**

**Figure 6:**
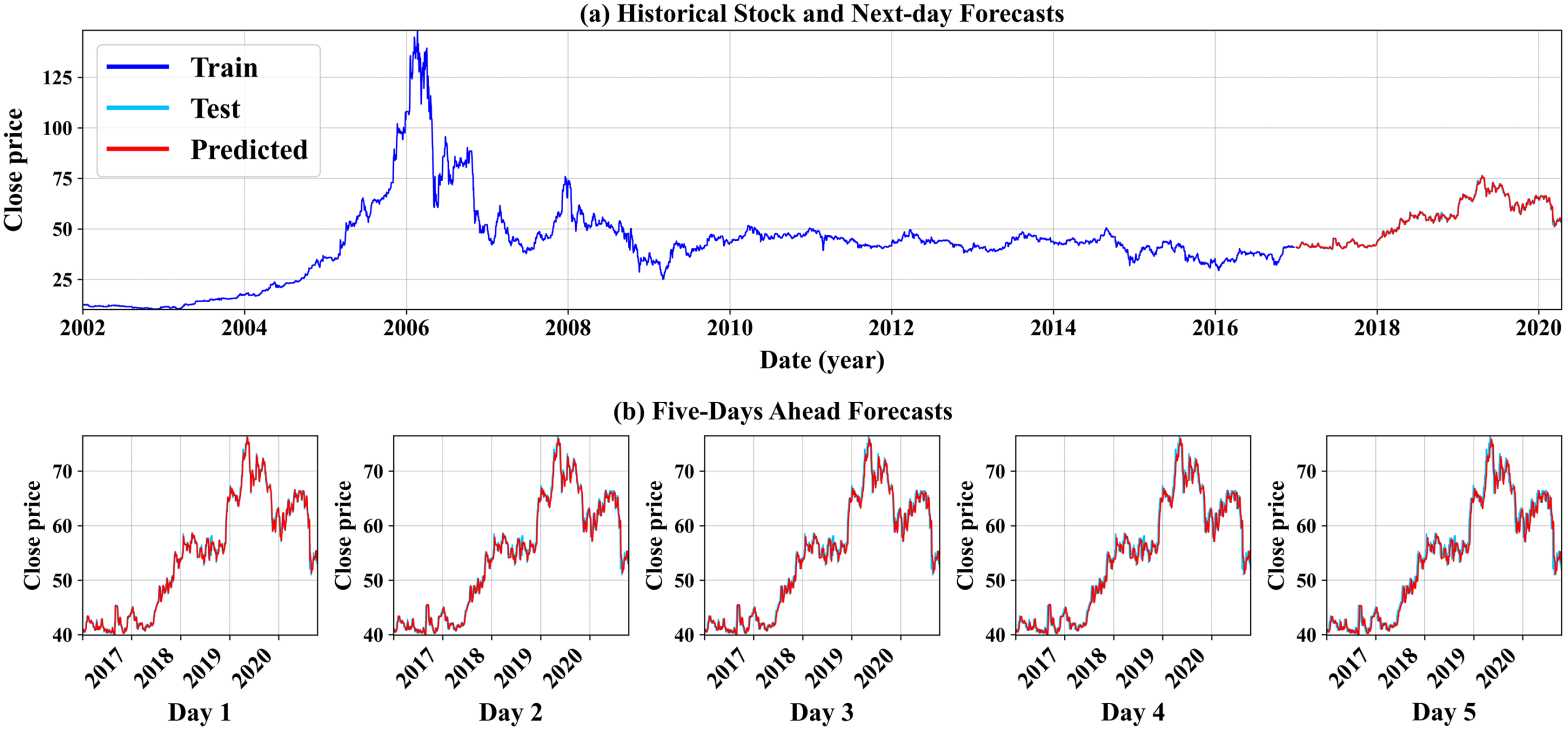
An illustration of the GRU model’s forecast results for Al Rajhi Bank.

As seen in Table 5, all results shown in this table are considered highly successful and effective in producing short-term forecasts. We can see that the experiment run on the preprocessed data conducted by the proposed feature engineering step (labeled by the * symbol) yields overall better forecasting results, with the best forecasting outcomes resulting from the GRU model (stressed in bold). We can conclude that the proposed methodology works best when paired with the GRU-based modeling option. This aligns with our initial intuition that RNN-based methods mostly lead to better forecasts than linear methods such as ARIMA and its variation. In addition, Fig. 6 demonstrates the GRU model’s forecasting results (GRU* only) for one company. The top subfigure (a) illustrates the historical daily stock price from the first-ever trading day until December 2016, followed by the next-day predictions using the unseen testing time series, from 01-01-2017 to the end of the company’s time series record (see. how the test and forecast estimates overlap due to the higher predictions’ precision). The bottom subfigure (b) shows the multistep closing price forecasts for the next five-days stock prices. We can also conclude that the model’s high precision predictions for the short-term forecasts, thus giving more insightful knowledge and better futuristic decision making. The forecasting illustrations of both the GRU model and the VARMAX model from the remaining nine companies selected in this experiment are shown in Figs. A2 and A3, respectively, and placed in the Appendix section.

## Conclusion

This article introduces an AI-enabled forecasting methodology consisting of a problem-tailored feature engineering and a well-architected GRU model design to yield short-term closing price forecasts of the Saudi stock market evaluated using a set of regression-based metrics and benchmarked with a baseline model, *i.e*., the VARMAX model. The experimental results based on the large-scale TASI index data are also presented and visually demonstrated. The empirical analysis revealed that our proposed forecasting methodology outperforms the most relevant existing studies to this work known to us by a significant margin. Besides the high precision results, it is due to the ability to generate highly accurate multi-step forecasts of the Saudi stock market for the next five business days, as opposed to only the next-day forecast conveyed by existing studies related to this work. In addition, this study demonstrates the successful power of the gated recurrent units (GRU) method in modeling historical multivariate-based stock market data. We plan to investigate further the improvement achieved by this work toward other related problems using a fine-grained (hourly time series) dataset with various feature selection strategies.

## Supplemental Information

10.7717/peerj-cs.1205/supp-1 Supplemental Information 1 Appendix.

10.7717/peerj-cs.1205/supp-2 Supplemental Information 2 Source code used to experiment this work.

10.7717/peerj-cs.1205/supp-3 Supplemental Information 3 The dataset used in this experiment.

## Additional Information and Declarations

### Data Availability

The following information was supplied regarding data availability: The raw data is available in the Supplemental Files. The data is also available at Kaggle:
https://www.kaggle.com/datasets/salwaalzahrani/saudi-stock-exchange-tadawul
Owner: Saudi Stock Exchange (Tadawul) Website: https://www.saudiexchange.sa/wps/portal/tadawul/home/

## References

[ref-1] Al-Shiab M (2006). The predictability of the amman stock exchange using the univariate autoregressive integrated moving average (ARIMA) model. Journal of Economic and Administrative Sciences.

[ref-2] Alamro R, McCarren A, Al-Rasheed A (2019). Predicting Saudi stock market index by incorporating GDELT using multivariate time series modelling.

[ref-3] Almasarweh M, Alwadi S (2018). ARIMA model in predicting banking stock market data. Modern Applied Science.

[ref-4] Alotaibi T, Nazir A, Alroobaea R, Alotibi M, Alsubeai F, Alghamdi A, Alsulimani T (2018). Saudi Arabia stock market prediction using neural network. International Journal on Computer Science and Engineering.

[ref-5] Alsubaie Y, El Hindi K, Alsalman H (2019). Cost-sensitive prediction of stock price direction: selection of technical indicators. IEEE Access.

[ref-6] Althelaya KA, El-Alfy E-SM, Mohammed S (2018). Evaluation of bidirectional LSTM for short-and long-term stock market prediction.

[ref-7] Ariyo AA, Adewumi AO, Ayo CK (2014). Stock price prediction using the ARIMA model.

[ref-8] Aseeri AO (2021). Uncertainty-aware deep learning-based cardiac arrhythmias classification model of electrocardiogram signals. Computers.

[ref-9] Banerjee D (2014). Forecasting of Indian stock market using time-series ARIMA model.

[ref-10] Bengio Y, Simard P, Frasconi P (1994). Learning long-term dependencies with gradient descent is difficult. IEEE Transactions on Neural Networks.

[ref-11] Bing Y, Hao JK, Zhang SC (2012). Stock market prediction using artificial neural networks. Advanced Engineering Forum.

[ref-12] Cakra YE, Trisedya BD (2015). Stock price prediction using linear regression based on sentiment analysis.

[ref-13] Cao J, Li Z, Li J (2019). Financial time series forecasting model based on CEEMDAN and LSTM. Physica A: Statistical Mechanics and Its Applications.

[ref-14] Chandar S, Sankar C, Vorontsov E, Kahou SE, Bengio Y (2019). Towards non-saturating recurrent units for modelling long-term dependencies. Proceedings of the AAAI Conference on Artificial Intelligence.

[ref-15] Chayama M, Hirata Y (2016). When univariate model-free time series prediction is better than multivariate. Physics Letters A.

[ref-16] Chen A-S, Leung MT, Daouk H (2003). Application of neural networks to an emerging financial market: forecasting and trading the Taiwan stock index. Computers & Operations Research.

[ref-17] Chen Y, Lin W, Wang JZ (2019). A dual-attention-based stock price trend prediction model with dual features. IEEE Access.

[ref-18] Chen K, Zhou Y, Dai F (2015). A LSTM-based method for stock returns prediction: a case study of China stock market.

[ref-19] Chung J, Gulcehre C, Cho K, Bengio Y (2014). Empirical evaluation of gated recurrent neural networks on sequence modeling. ArXiv preprint.

[ref-20] Dey R, Salem FM (2017). Gate-variants of gated recurrent unit (GRU) neural networks.

[ref-21] Dozat T (2016). Incorporating nesterov momentum into adam. Proceedings of 4th International Conference on Learning Representations, Workshop Track 2016.

[ref-22] Ehlers J, Way R (2010). Zero lag (well, almost). https://www.mesasoftware.com/papers/ZeroLag.pdf.

[ref-23] Elman JL (1990). Finding structure in time. Cognitive Science.

[ref-24] Fama EF (1995). Random walks in stock market prices. Financial Analysts Journal.

[ref-25] Gharehchopogh FS, Bonab TH, Khaze SR (2013). A linear regression approach to prediction of stock market trading volume: a case study. International Journal of Managing Value and Supply Chains.

[ref-26] Gilmore CG, McManus GM (2003). Random-walk and efficiency tests of central European equity markets. Managerial Finance.

[ref-27] Guresen E, Kayakutlu G, Daim TU (2011). Using artificial neural network models in stock market index prediction. Expert Systems with Applications.

[ref-28] Hochreiter S, Schmidhuber J (1997). Long short-term memory. Neural Computation.

[ref-29] Hull A (2005). How to reduce lag in a moving average. https://alanhull.com/hull-moving-average.

[ref-30] Jarrah M, Salim N (2019). A recurrent neural network and a discrete wavelet transform to predict the Saudi stock price trends. International Journal of Advanced Computer Science and Applications.

[ref-31] Jarrett JE (2008). Random walk, capital market efficiency and predicting stock returns for Hong Kong exchanges and clearing limited. Management Research News.

[ref-32] Jarrett JE, Kyper E (2011). ARIMA modeling with intervention to forecast and analyze Chinese stock prices. International Journal of Engineering Business Management.

[ref-33] Javed Awan M, Mohd Rahim MS, Nobanee H, Munawar A, Yasin A, Zain AM (2021). Social media and stock market prediction: a big data approach. Computers, Materials & Continua.

[ref-34] Junior PR, Salomon FLR, de Oliveira Pamplona E (2014). ARIMA: an applied time series forecasting model for the bovespa stock index. Applied Mathematics.

[ref-35] Kadiyala A, Kumar A (2014). Vector time series models for prediction of air quality inside a public transportation bus using available software. Environmental Progress & Sustainable Energy.

[ref-36] Kara Y, Boyacioglu MA, Baykan ÖK (2011). Predicting direction of stock price index movement using artificial neural networks and support vector machines: the sample of the Istanbul stock exchange. Expert Systems with Applications.

[ref-37] Lang K, Zhang M, Yuan Y, Yue X (2019). Short-term load forecasting based on multivariate time series prediction and weighted neural network with random weights and kernels. Cluster Computing.

[ref-38] Lawler GF, Limic V (2010). Random walk: a modern introduction.

[ref-39] LeCun Y, Bengio Y (1998). Convolutional networks for images, speech, and time series. The Handbook of Brain Theory and Neural Networks.

[ref-40] Li H, Dagli CH, Enke D (2007). Short-term stock market timing prediction under reinforcement learning schemes.

[ref-41] Li X, Wang C, Dong J, Wang F, Deng X, Zhu S (2011). Improving stock market prediction by integrating both market news and stock prices.

[ref-42] McCulloch WS, Pitts W (1943). A logical calculus of the ideas immanent in nervous activity. The Bulletin of Mathematical Biophysics.

[ref-43] Mehmood MS, Mehmood A, Mujtaba BG (2012). Stock market prices follow the random walks: evidence from the efficiency of Karachi stock exchange. European Journal of Economics, Finance and Administrative Sciences.

[ref-44] Mekayel Anik M, Shamsul Arefin M, Ali Akber Dewan M (2020). An intelligent technique for stock market prediction.

[ref-45] Mizuno H, Kosaka M, Yajima H, Komoda N (1998). Application of neural network to technical analysis of stock market prediction. Studies in Informatic and Control.

[ref-46] Olah C (2015). Understanding LSTM networks. http://colah.github.io/posts/2015-08-Understanding-LSTMs.

[ref-66] Östermark R (1994). Using neural nets in modelling vector time series. Kybernetes.

[ref-47] Pang X, Zhou Y, Wang P, Lin W, Chang V (2020). An innovative neural network approach for stock market prediction. The Journal of Supercomputing.

[ref-48] Pawar K, Jalem RS, Tiwari V (2019). Stock market price prediction using LSTM RNN. Emerging Trends in Expert Applications and Security.

[ref-49] Perry MB (2010). The weighted moving average technique.

[ref-50] Rozario E, Holt S, West J, Ng S (2020). A decade of evidence of trend following investing in cryptocurrencies. ArXiv preprint.

[ref-51] SAS (2016). The varmax procedure. https://bit.ly/3ACCkuy.

[ref-52] Schäfer AM, Zimmermann HG (2006). Recurrent neural networks are universal approximators.

[ref-53] Seber GA, Lee AJ (2012). Linear regression analysis.

[ref-54] Selvamuthu D, Kumar V, Mishra A (2019). Indian stock market prediction using artificial neural networks on tick data. Financial Innovation.

[ref-55] Selvin S, Vinayakumar R, Gopalakrishnan E, Menon VK, Soman K (2017). Stock price prediction using LSTM, RNN and CNN-sliding window model.

[ref-56] Shen S, Shen Y (2016). ARIMA model in the application of Shanghai and Shenzhen stock index. Applied Mathematics.

[ref-57] Siew HL, Nordin MJ (2012). Regression techniques for the prediction of stock price trend.

[ref-58] Smith TG (2017). pmdarima: ARIMA estimators for Python. http://www.alkaline-ml.com/pmdarima.

[ref-59] Sun J, Xiao K, Liu C, Zhou W, Xiong H (2019). Exploiting intra-day patterns for market shock prediction: a machine learning approach. Expert Systems with Applications.

[ref-60] TASI (2020). Saudi stock market index (tasi) dataset. https://www.kaggle.com/datasets/salwaalzahrani/saudi-stock-exchange-tadawul.

[ref-61] Wadi S, Almasarweh M, Alsaraireh AA, Aqaba J (2018). Predicting closed price time series data using ARIMA model. Modern Applied Science.

[ref-62] Yetis Y, Kaplan H, Jamshidi M (2014). Stock market prediction by using artificial neural network.

[ref-63] Yin Y, Shang P (2016). Forecasting traffic time series with multivariate predicting method. Applied Mathematics and Computation.

[ref-64] Zhang GP (2003). Time series forecasting using a hybrid ARIMA and neural network model. Neurocomputing.

[ref-65] Zhang Y, Zhong M, Geng N, Jiang Y (2017). Forecasting electric vehicles sales with univariate and multivariate time series models: the case of China. PLOS ONE.

