# Supplementary material for: Effective short-term forecasts of Saudi stock price trends using technical indicators and large-scale multivariate time series
Source: PeerJ Comput Sci. 2023 Jan 6;9:e1205. doi: 10.7717/peerj-cs.1205 (PMC10280263; doi:10.7717/peerj-cs.1205)
Supplement: Supplemental Information 1 [file peerj-cs-09-1205-s001.pdf]

A APPENDIX

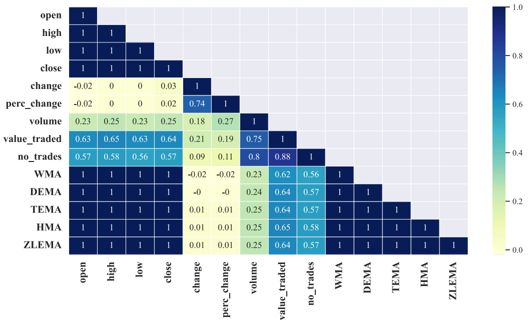

(a) Saudi Arabia Refineries Co.

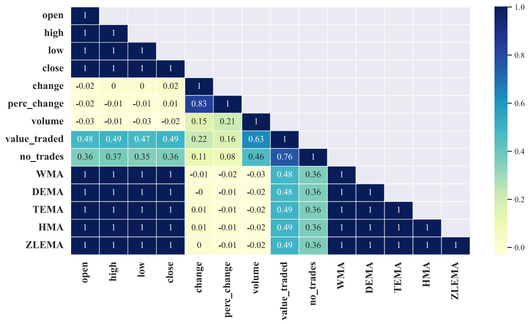

(b) National Industrialization Co.

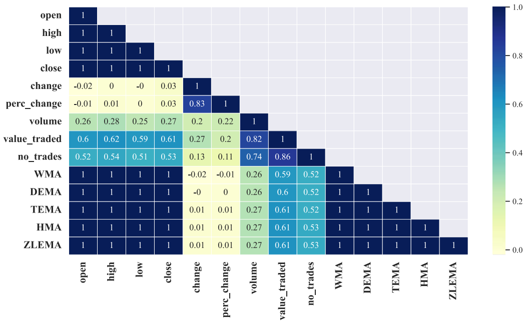

(c) SPIMACO Co.

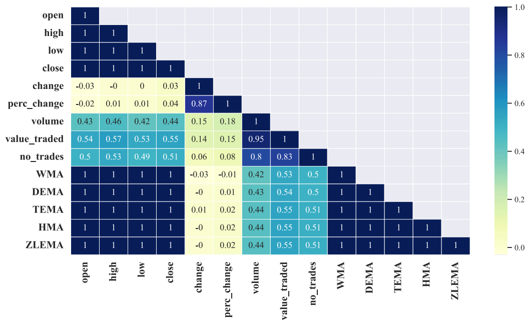

(d) Saudi Electricity Co.

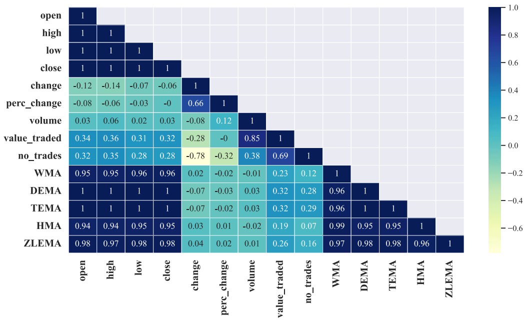

(e) Emaar The Economic City

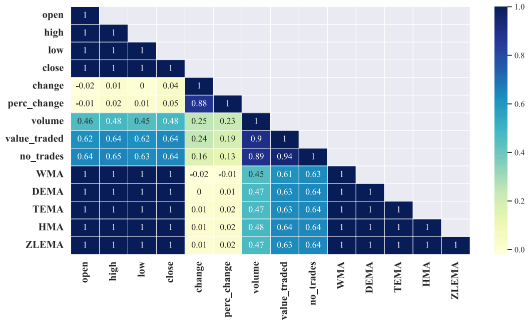

(f) Fitaihi Holding Group

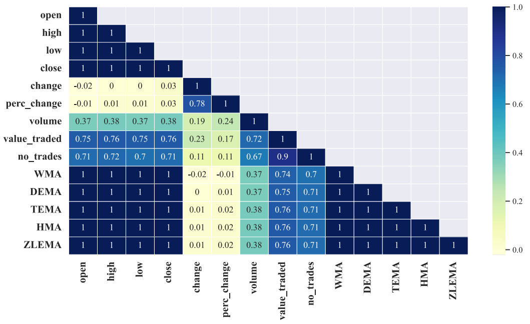

(g) Al-Ahsa Development Co.

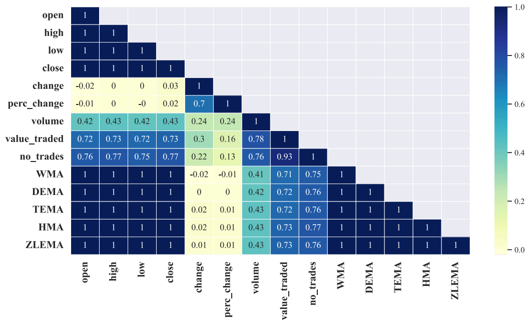

(h) Al Gassim Investment Holding Co.

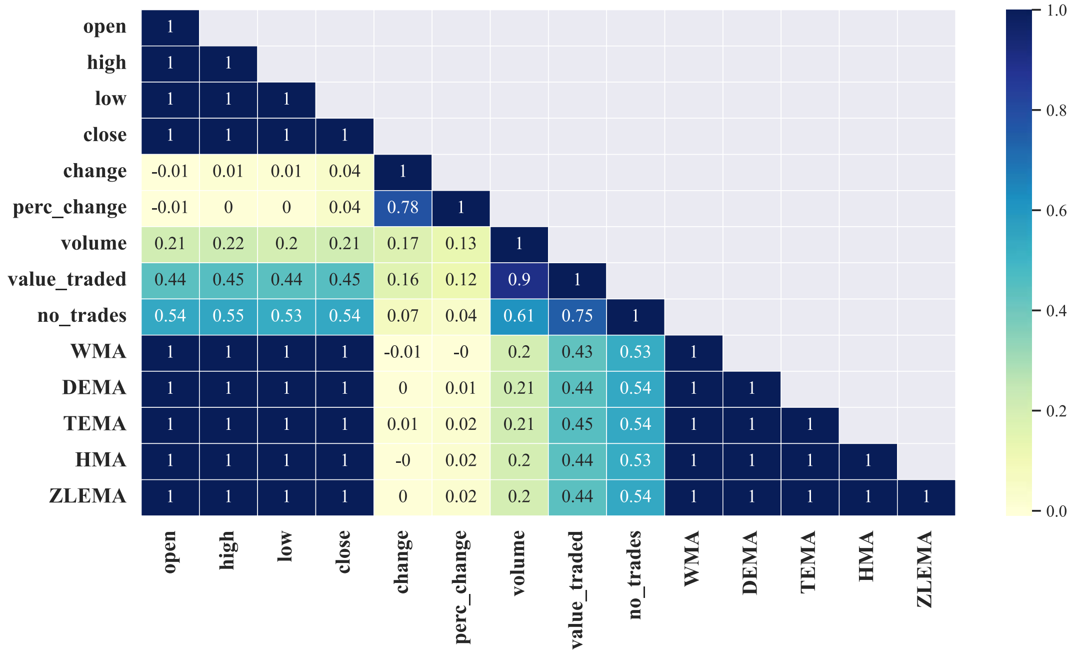

(i) Saudi Telecom Co.

Figure A.1. Illustration of the correlation matrices applied on the selected nine companies involved in this experiment.

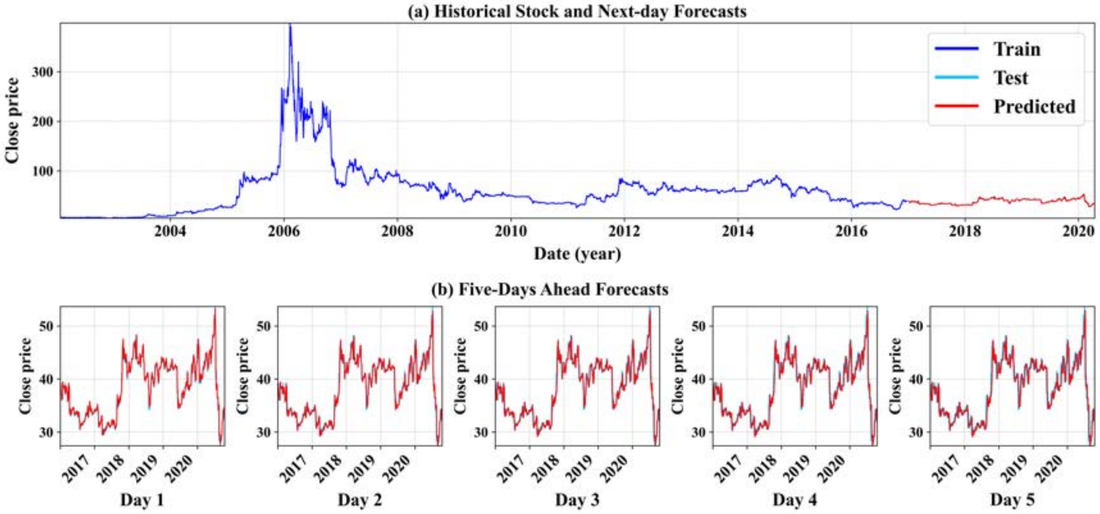

(a) Saudi Arabia Refineries Co.

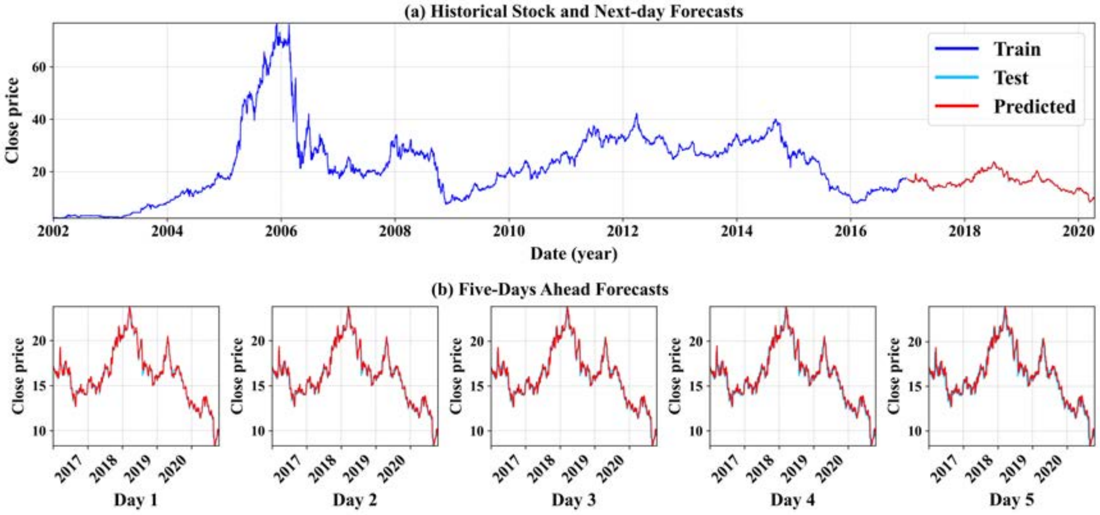

(b) National Industrialization Co.

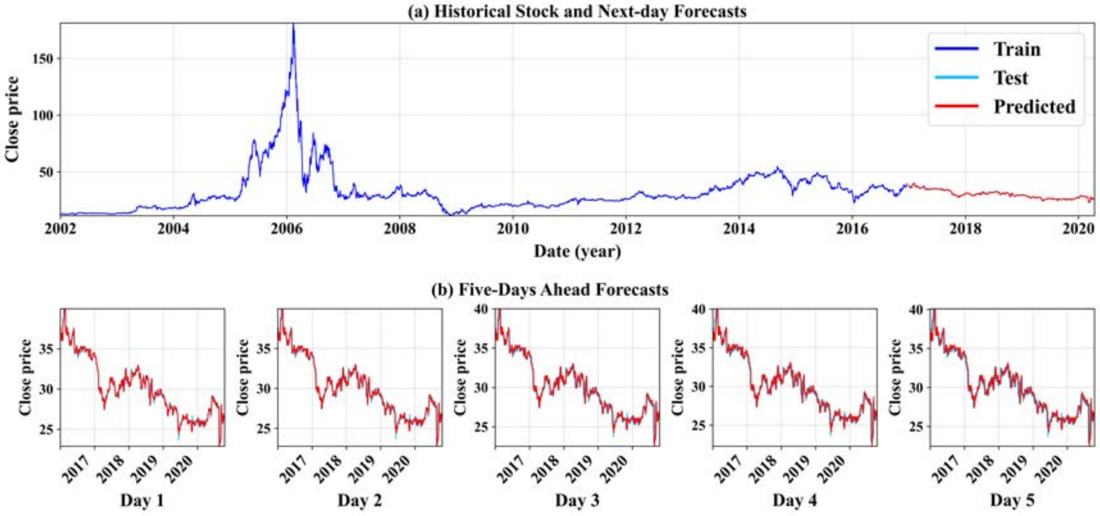

(c) SPIMACO CO.

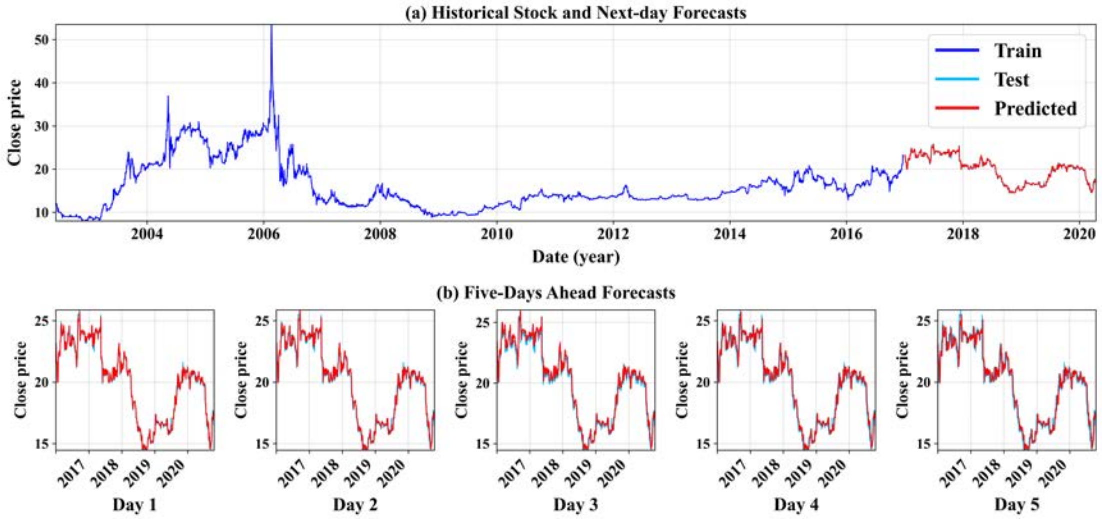

(d) Saudi Electricity Co.

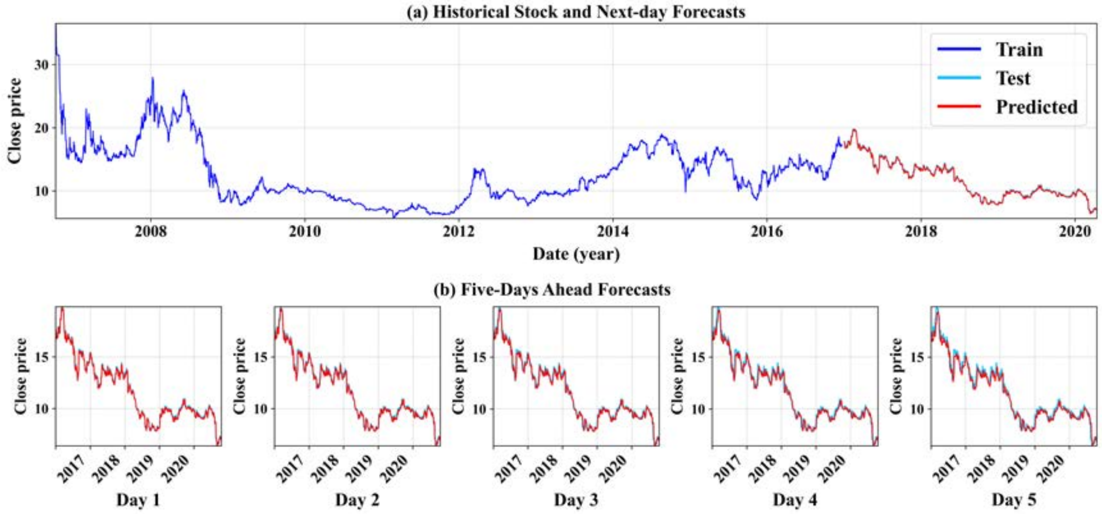

(e) Emaar The Economic City

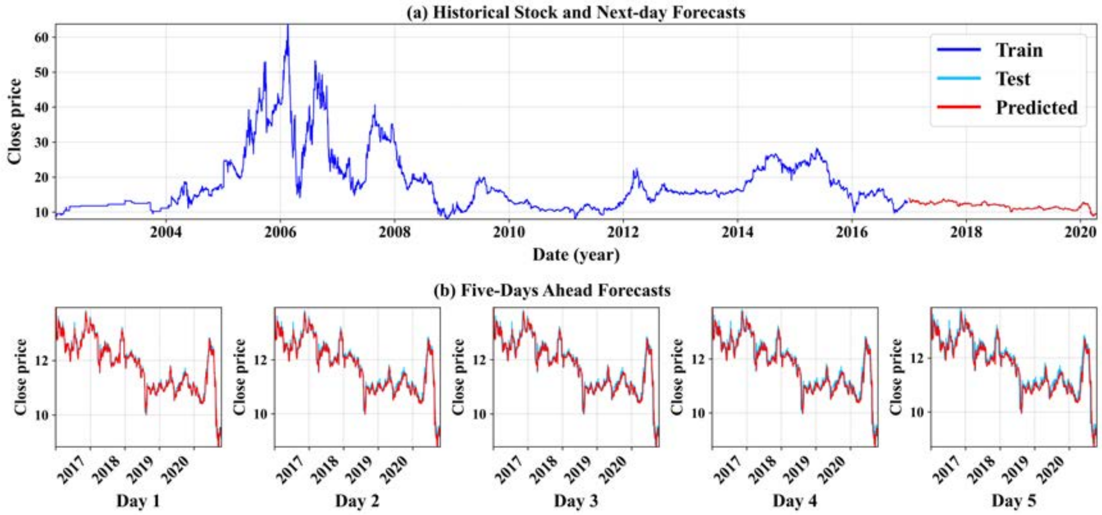

(f) Fitaihi Holding Group

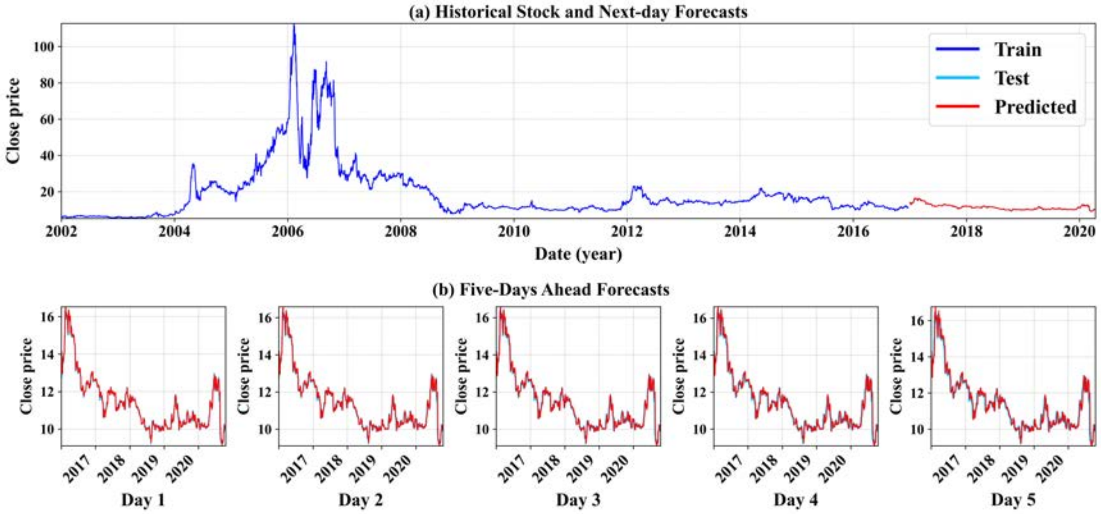

(g) Al-Ahsa Development Co.

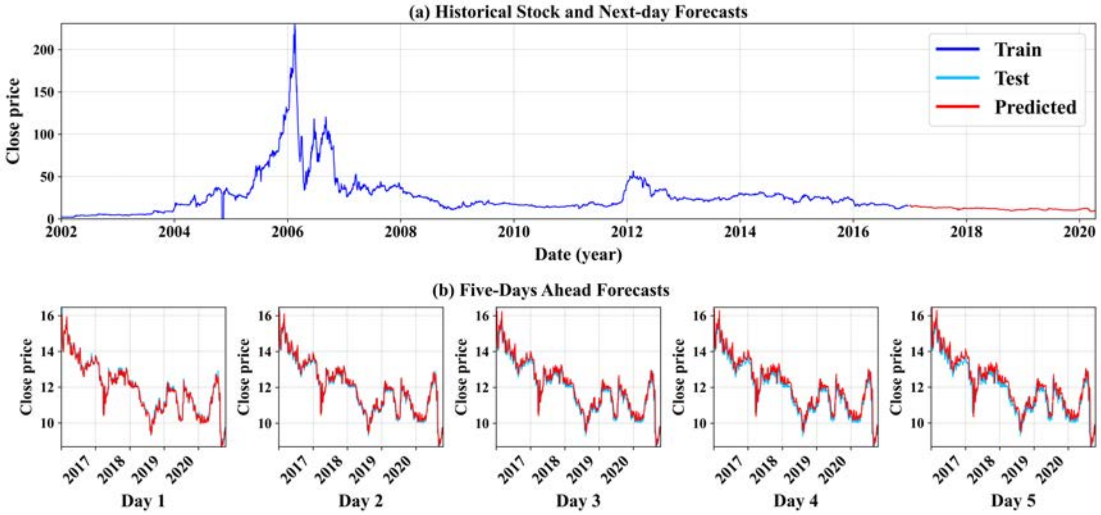

(h) Al Gassim Investment Holding Co.

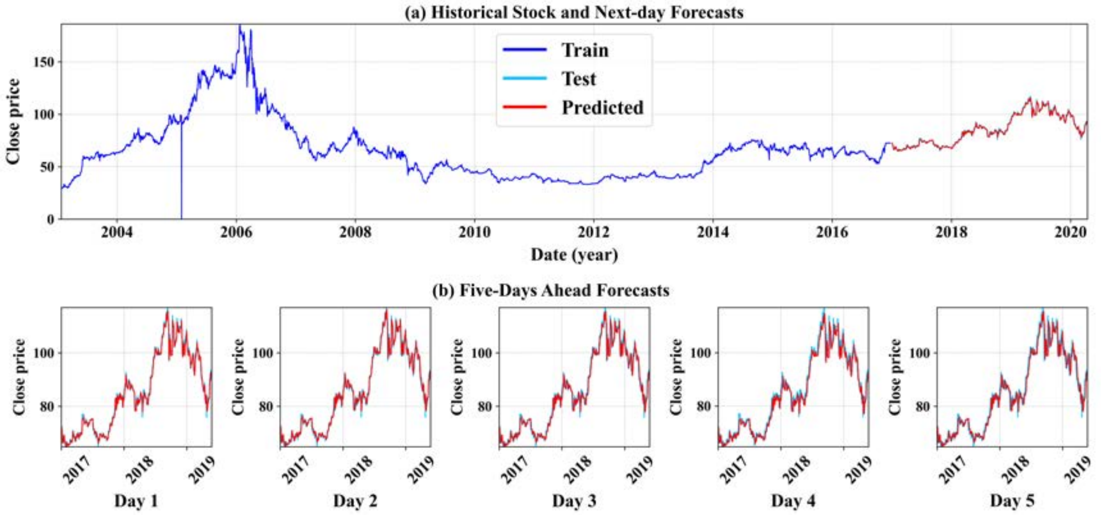

(i) Saudi Telecom Co.

Figure A.2. Forecasting results of the proposed GRU model from the nine companies selected in this experiment.

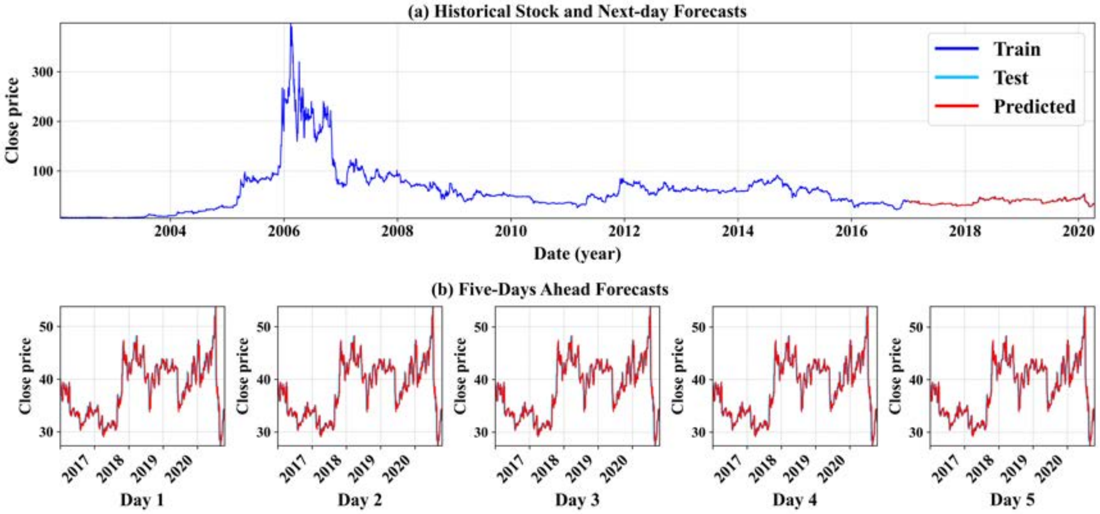

(a) Saudi Arabia Refineries Co.

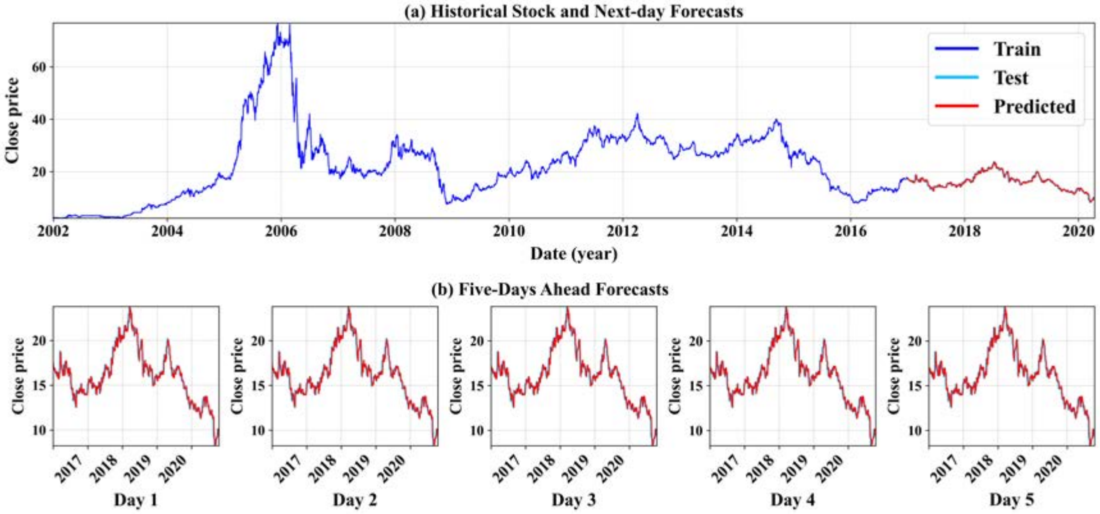

(b) National Industrialization Co.

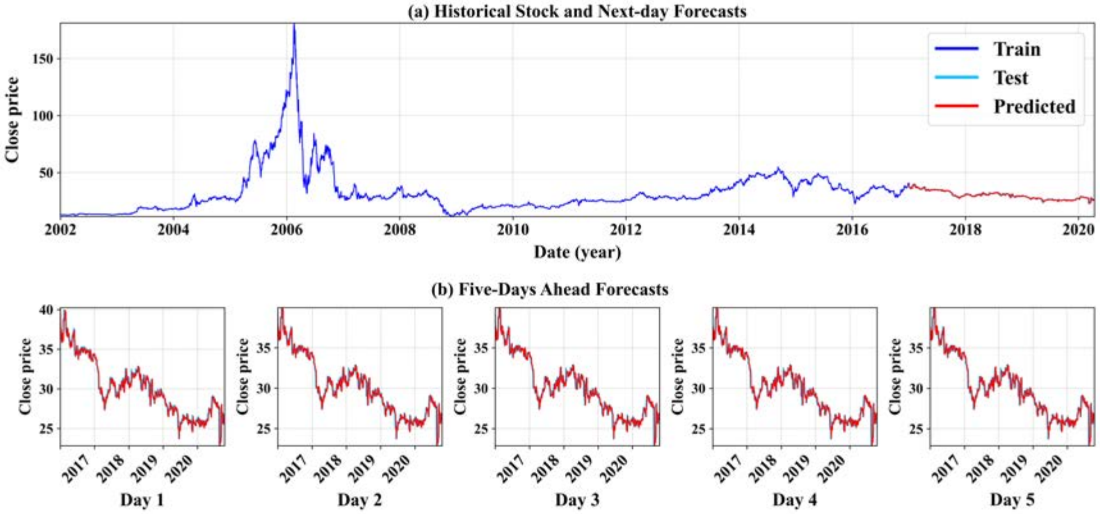

(c) SPIMACO CO.

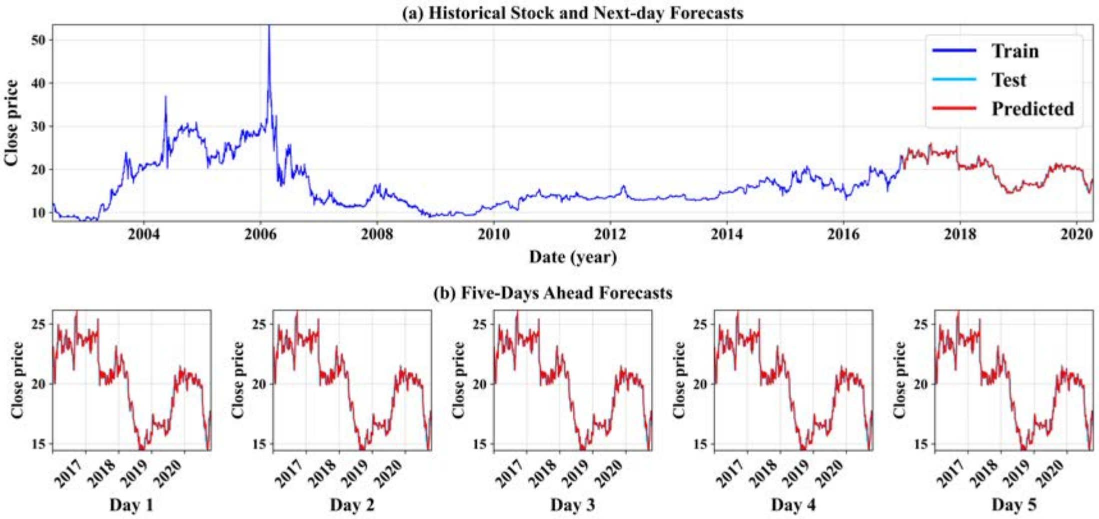

(d) Saudi Electricity Co.

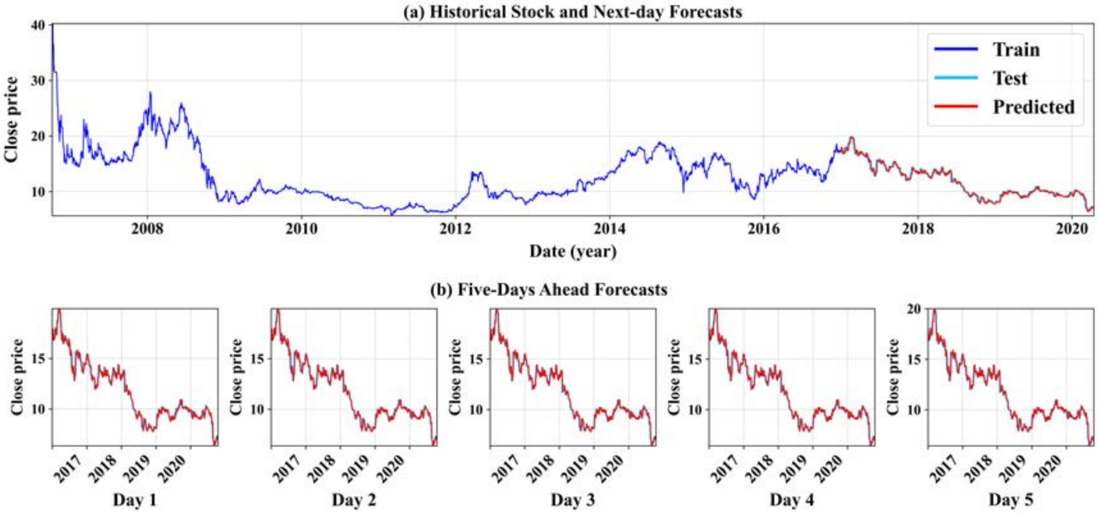

(e) Emaar The Economic City

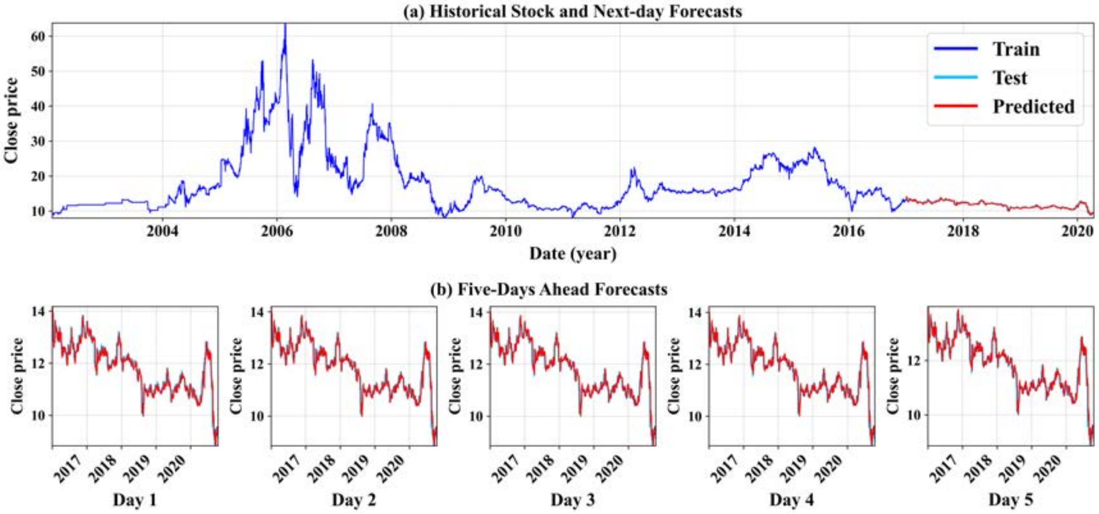

(f) Fitaihi Holding Group

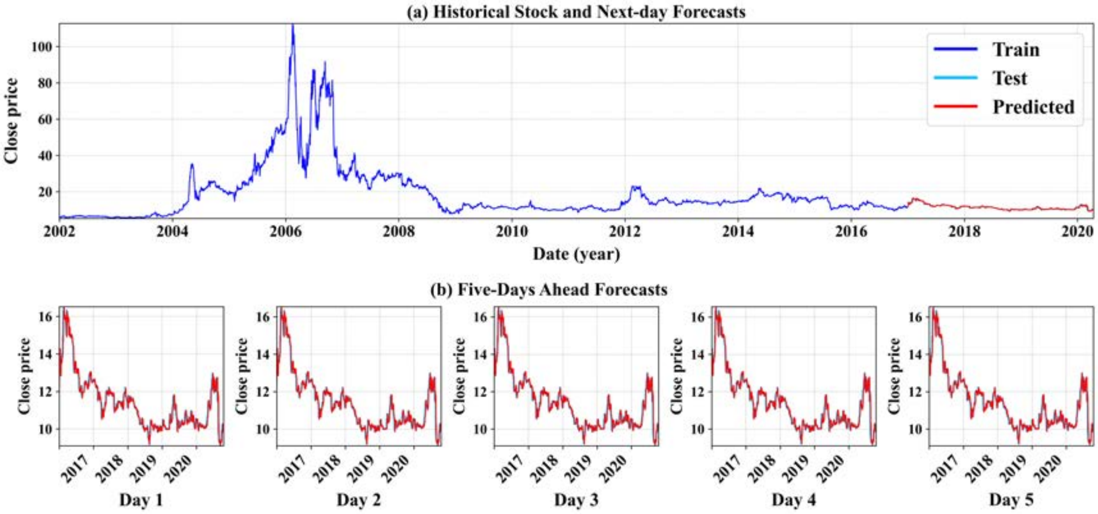

(g) Al-Ahsa Development Co.

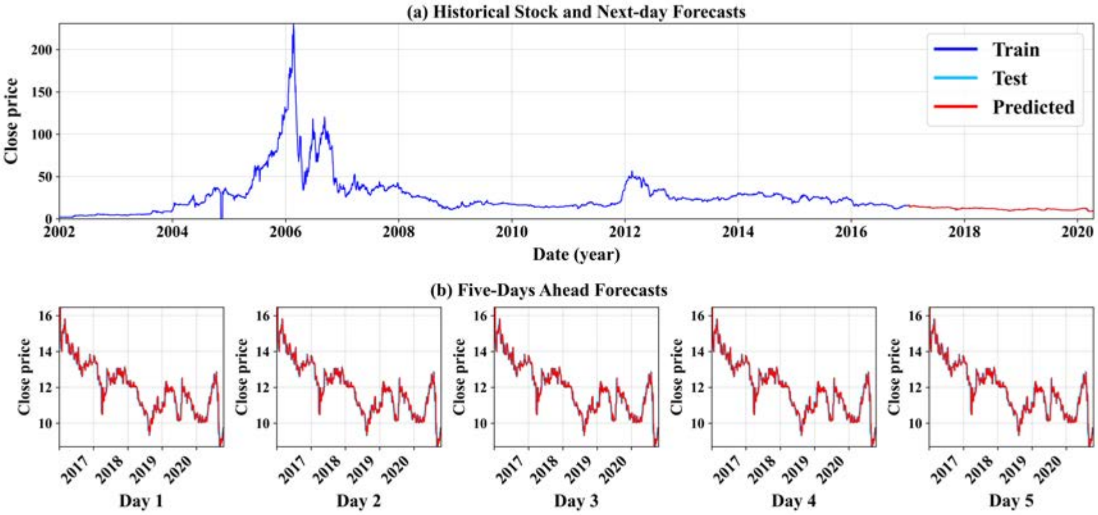

(h) Al Gassim Investment Holding Co.

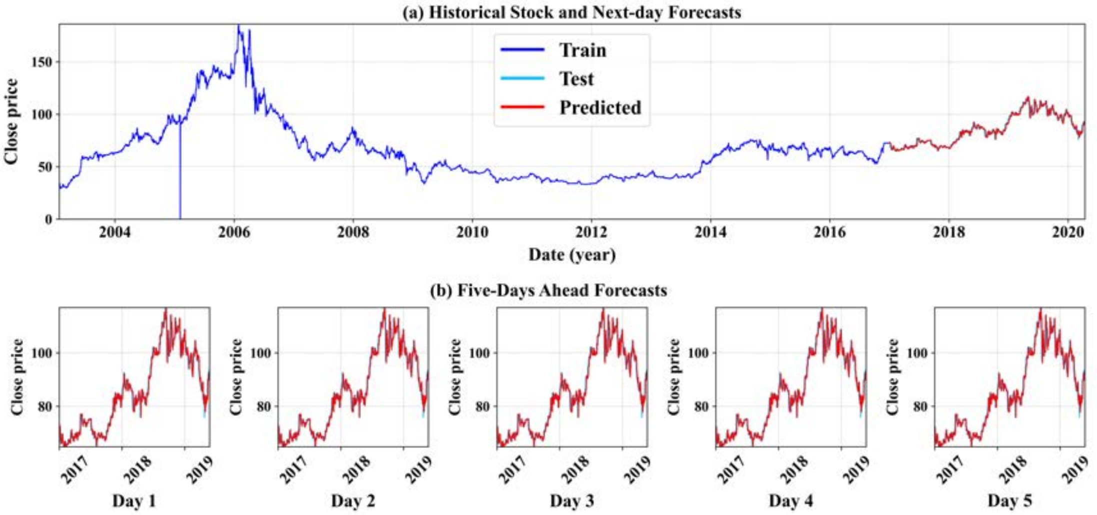

(i) Saudi Telecom Co.

Figure A.3. Forecasting results of the baseline VARMAX model from the nine companies selected in this experiment.
